# Conversion‐Type Anodes: Challenges, Mitigation Strategies, and Evaluation Guidelines

**DOI:** 10.1002/smll.75242

**Published:** 2026-08-17

**Authors:** Sunhyun Hwang, Won‐Sub Yoon

**Affiliations:** ^1^ Department of Energy Science Sungkyunkwan University Suwon Republic of Korea; ^2^ SKKU Institute of Energy Science and Technology (SIEST) Sungkyunkwan University Suwon Republic of Korea

**Keywords:** challenges, conversion‐type anode, evaluation guidelines, Lithium‐ion battery, mitigation strategies

## Abstract

Conversion‐type anode materials enable multi‐electron redox reactions and therefore offer theoretical capacities beyond those of classical insertion‐based anode materials such as graphite. However, their practical implementation is limited by multiple coupled penalties that evolve with cycling and electrode design. This review organizes recent progress in conversion‐type anodes (including transition metal oxides, sulfides, selenides, and phosphides) around five recurring limitations: (i) voltage hysteresis and low round‐trip energy efficiency, (ii) low initial Coulombic efficiency and cyclable‐lithium inventory loss, (iii) interphase instability with continuous electrolyte reduction and dissolution‐mediated cross‐talk, (iv) chemo‐mechanical damage leading to fracture, contact loss, and conductive network degradation, and (v) transport and accessibility limitations that intensify in thick, high‐areal‐loading electrodes under practical electrolyte amounts. We survey mitigation strategies spanning active‐material and microstructure design, composite and processing controls, electrolyte design and formation protocols, and electrode‐architecture design to improve ionic accessibility and reduce concentration polarization in thick electrodes. To reduce mechanistic overinterpretation, this review develops a coupled‐penalty evaluation framework in which each section closes with an integrated interpretation and a validation checklist defining the evidence required for mechanistic claims. Overall, this review provides practical guidelines for distinguishing intrinsic conversion/reconversion pathway changes from electrode‐level accessibility, resistance, interphase, and formation‐history effects in conversion‐type anodes.

## Graphical abstract

Conversion‐type anodes offer high theoretical capacity but exhibit coupled electrochemical, interfacial, mechanical, and transport limitations that complicate performance interpretation. A practical evaluation framework is presented to distinguish intrinsic reaction chemistry from electrode‐scale effects through complementary diagnostics and standardized testing. This framework enables more reliable mechanistic interpretation and supports the development of practical high‐energy lithium‐ion batteries.

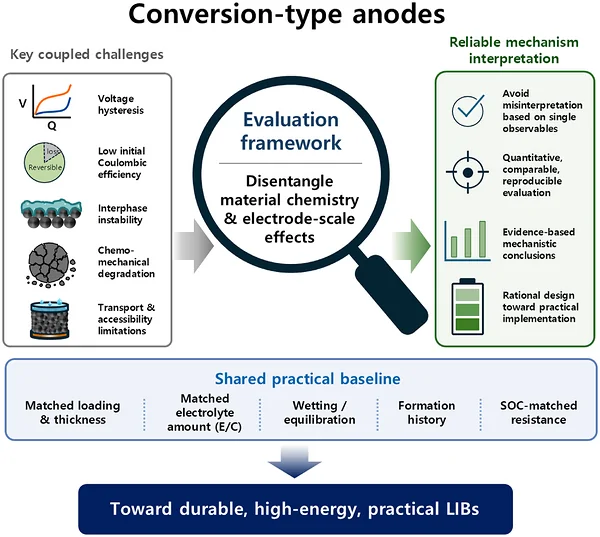

## 1 Introduction

Conversion‐type anodes remain attractive for next‐generation Li‐ion batteries because multi‐electron redox reactions enable capacities beyond those of graphite and classical intercalation anodes [1, 2]. However, practical application is constrained by poor cyclability, low Coulombic efficiency (CE), interfacial instability, and microstructural evolution rather than by capacity alone [3, 4]. Following the initial demonstration of reversible conversion in nanosized transition metal oxides [5], subsequent reviews further consolidated the field by summarizing conversion‐based anodes for lithium‐ion batteries (LIBs) and sodium‐ion batteries (SIBs), recent advances in conversion‐type electrodes for post‐LIBs, and the remaining challenges of conversion‐type anodes discussed within broader surveys of LIB and SIB anode materials [6, 7, 8, 9, 10, 11, 12, 13]. These reviews have been essential for cataloging conversion‐type materials, reaction mechanisms, and mitigation strategies. However, many prior discussions are organized primarily by material class, reaction chemistry, or individual degradation mode, whereas practical interpretation often fails because several penalties evolve simultaneously in composite electrodes. The distinct contribution of this review is therefore not the identification of each penalty as a new degradation mode, but the organization of these penalties into a coupled‐penalty evaluation framework for practical LIB conversion‐type anodes. This framework asks whether apparent improvements in capacity, voltage hysteresis, initial Coulombic efficiency (ICE), interphase stability, mechanical durability, or rate capability can be assigned to intrinsic conversion/reconversion chemistry, or whether they arise from electrode‐level accessibility, resistance, wetting, formation history, or utilization effects. The present review builds on the existing literature but focuses specifically on practical LIB conversion‐type anodes, with emphasis on coupled penalties and the minimum evidence required for mechanistic interpretation under realistic electrode and electrolyte conditions, rather than on the broader anode‐side challenges covered in adjacent reviews of emerging battery configurations [14].

A central challenge is that the major penalties rarely appear in isolation. Voltage hysteresis, low ICE, dynamic interphases, chemo‐mechanical degradation, and transport/accessibility limitations co‐evolve and can obscure causality, so similar electrochemical responses may arise from differences in accessibility or electrode polarization rather than from reduced intrinsic conversion barriers [15, 16, 17, 18, 19, 20, 21]. More broadly, coupled interphase evolution, mechanical damage, and transport limitations recur across rechargeable‐battery chemistries, underscoring the need for causality‐preserving protocols when comparing mitigation strategies [22, 23].

**FIGURE 1:**
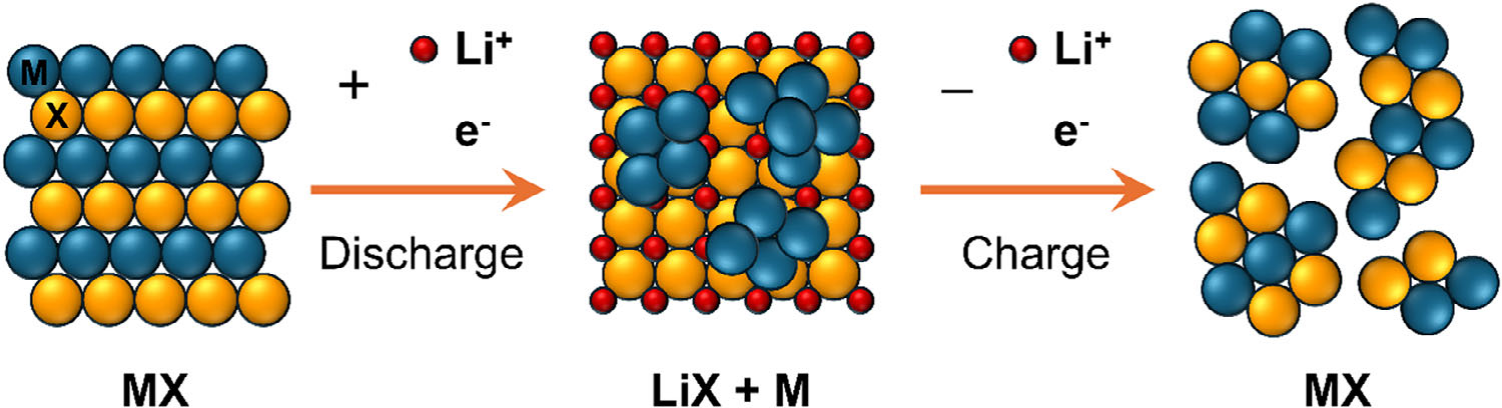
Representative conversion–reconversion process in a conversion‐type anode.

Accordingly, this review organizes recent progress around these five practically dominant limitations and surveys mitigation strategies spanning active‐material/microstructure design, conductive frameworks and buffering matrices, binder and processing choices, electrolyte design and formation protocols, and electrode architectures designed to improve ionic and electronic transport in high‐loading anodes. To make the evaluation methodology explicit, the review uses three connected elements: a shared practical baseline for comparison, cross‐coupling logic among the five penalties, and penalty‐specific diagnostic checks. To reduce redundancy while preventing misattribution, the shared practical baseline for comparison is defined once in Section 2, and the later validation checklists focus on penalty‐specific diagnostics rather than repeatedly restating the same baseline criteria. Each section closes with an integrated interpretation and a short validation checklist that flags the minimum reporting variables and diagnostics needed to support mechanistic claims under practical constraints. Where resistance evolution is discussed, interpretation follows impedance measured at matched state of charge (SOC) under a transparent protocol, or another resistance‐related measurement obtained under equivalent conditions, such as direct current internal resistance (DC‐IR), overpotential, or polarization [24, 25]. Figure 1 summarizes the generic conversion–reconversion framework underlying the coupled penalties discussed in this review.

During lithiation, the parent compound is transformed into a metal–LiX nanocomposite through intermediate reconstructed states (M = transition metal species; X = O, S, Se, or P). During delithiation, reconversion proceeds from this highly dispersed metal–LiX matrix and need not retrace the forward sequence or directly regenerate the equilibrium parent phase, MX. This schematic provides the mechanistic starting point for the coupled penalties discussed in this review, including voltage hysteresis, incomplete first‐charge recovery, dynamic interphase evolution, chemo‐mechanical damage, and thick‐electrode transport limitations.

## 2 Scope and Coupling Premise

### 2.1 Scope of this Review

This review focuses on conversion‐type anodes for Li‐ion batteries, including representative transition metal oxides, sulfides, selenides, and phosphides. The emphasis is on composite‐electrode behavior under high areal loading, electrode density, and thickness relevant to practical cells, and limited electrolyte availability, rather than on idealized thin, electrolyte‐rich half‐cell tests. The discussion, therefore, prioritizes the consequences of conversion chemistry in composite electrodes, including voltage hysteresis, cyclable lithium loss, interphase instability, chemo‐mechanical degradation, and transport/accessibility limitations, along with the mitigation strategies used to alleviate them under practical conditions.

Composite Li metal anodes and related Li‐metal‐based composite architectures are important adjacent anode systems, but they are not included as a primary material class in this review because their dominant storage process is not the conversion–reconversion formation of metal–LiX nanocomposites used here to define conversion‐type anodes. Nevertheless, several evaluation principles discussed below, particularly those concerning interphase evolution, mechanical instability, electrolyte availability, and practical cell‐level benchmarking, are conceptually relevant to Li‐metal‐based composite anodes.

Because much of the conversion‐anode literature was obtained under thin‐electrode, electrolyte‐rich, or lightly loaded half‐cell conditions, the cited examples are not treated uniformly as practical validation. Instead, this review distinguishes the evidence role of each example. Studies performed under idealized laboratory conditions are used mainly as mechanistic or proof‐of‐concept evidence, whereas practical relevance requires additional reporting of loading/thickness/density, electrolyte amount or electrolyte‐to‐capacity ratio (E/C), wetting/equilibration history, formation protocol, full‐cell context, and resistance or utilization diagnostics. Table 1 summarizes this evidence‐context distinction for representative conversion‐anode strategies.

**TABLE 1:** Representative conversion‐anode strategies, evidence context, and practical translation checks. The table is intended to clarify how representative examples are used in this review. Strategies demonstrated mainly in thin, electrolyte‐rich, or lightly loaded half cells are treated as mechanistic or proof‐of‐concept evidence unless practical descriptors such as loading/thickness/density, electrolyte amount or E/C, wetting/equilibration, formation history, full‐cell context, and resistance/utilization diagnostics are reported. E/C denotes the electrolyte‐to‐capacity ratio.

Material/strategy class	Representative examples discussed	Main claimed benefit	Evidence context in this review	Practical interpretation and translation checks
Nanostructured and hollow oxide conversion anodes	Yolk‐shell NiO, carbon‐coated yolk‐shell V_2_O_3_, multilayer CuO@NiO hollow spheres, hollow Co_3_O_4_ [58, 59, 60, 61]	Shortened transport length, strain accommodation, improved rate/cycling response	Mostly treated as proof‐of‐concept or mechanistic evidence for nanoscale confinement and strain buffering	Practical validation requires loading/thickness/density, E/C ratio, wetting/equilibration, ICE/CE, resistance evolution, and full‐cell or lean‐electrolyte testing
Carbon‐confined and conductive‐framework composites	Fe_3_O_4_/Cu nano‐architectures, Fe_3_O_4_@Fe_3_C–C, Co_2_P quantum‐dot/carbon hybrids, CoS_2_/graphene/CNT aerogels [50, 53, 54, 55, 56, 57]	Improved electronic percolation, reduced polarization, contact preservation	Useful for identifying conductive‐network design principles, but often demonstrated in laboratory half cells	Claims should be supported by conductivity/CBD evidence, SOC‐matched resistance, areal loading, electrode density, and utilization‐uniformity diagnostics
Sulfide, selenide, and phosphide conversion anodes	CuS, MoS_2_, CoP, FeP, Co_2_P, one‐dimensional chalcogenides [45, 46, 47, 48, 49, 50, 51, 52]	Altered reaction pathway, improved kinetics, enhanced reversible conversion	Provides broader chemical scope beyond oxides; practical relevance depends strongly on electrode formulation and electrolyte compatibility	Translation requires CE/ICE, dissolution or shuttle checks where relevant, electrolyte compatibility, and thick‐electrode/full‐cell validation
Electrolyte, interphase, and formation strategies	Fluoroethylene carbonate‐containing electrolytes, artificial interphase/coating strategies, formation‐protocol studies [37, 38, 39, 40, 41, 42, 43, 44]	Suppressed continuous electrolyte reduction and stabilized resistance growth	Evidence is highly protocol‐ and cell‐context‐dependent	Evaluate using time‐resolved CE, matched‐SOC impedance, interphase tracking across cycling, and explicitly reported wetting/formation conditions
Lithium‐inventory management	Prelithiation and sacrificial lithium‐source strategies [33, 34, 35, 36]	Recovery of deliverable full‐cell capacity after first‐cycle lithium loss	Treated as inventory compensation rather than intrinsic ICE improvement	Report supplied Li in mAh cm^−^^2^ relative to anode irreversible capacity, N/P ratio, post‐prelithiation CE, and resistance evolution
Thick‐electrode architecture, wetting, and utilization diagnostics	Operando reaction localization, electrolyte infiltration modeling/visualization, tortuosity‐engineered architectures [26, 27, 28, 29, 30, 31, 32]	Improved interpretation of transport/accessibility limitation under practical geometry	Provides direct diagnostic evidence for thick‐electrode translation and utilization nonuniformity	Require thickness/loading series, transport descriptors, E/C and wetting/equilibration reporting, reaction‐distribution maps, and SOC‐matched resistance

### 2.2 Working Definitions and Interpretive Conventions Used in this Review

For clarity, the following terms and conventions are used throughout this review.

#### 2.2.1 Conversion‐Type Anodes

Anodes whose reversible capacity relies primarily on the formation and decomposition of metal–LiX nanocomposites during cycling [4, 5].

#### 2.2.2 Voltage Hysteresis and Round‐Trip Energy Efficiency

In this review, voltage hysteresis refers to the separation between the lithiation and delithiation voltages, whereas round‐trip energy efficiency denotes the ratio of discharge energy to charge energy. The two are treated separately because voltage hysteresis can narrow without improving round‐trip energy efficiency when polarization is redistributed rather than reduced.

#### 2.2.3 Lithium Inventory and Initial Coulombic Efficiency

Initial Coulombic efficiency (ICE) is the ratio of the first charge to the first discharge capacity. Here, ICE is interpreted as a full‐cell lithium‐inventory penalty, since first‐cycle lithium loss must ultimately be compensated by cathode oversizing or lithium supplementation in full cells [36, 62].

#### 2.2.4 Electrolyte Availability and Accessibility

Electrolyte availability is the amount of electrolyte available to the cell, reported here as the electrolyte‐to‐capacity ratio (E/C). Wetting refers to the extent of electrolyte infiltration and equilibration achieved under a specified protocol [17, 63]. In this review, accessibility denotes the fraction of the electrode that is effectively contacted by electrolyte and thus available for ionic transport under a given wetting state.

#### 2.2.5 Intrinsic Material‐Level Kinetics

In this review, intrinsic material‐level kinetics refer to active‐material processes that control the local conversion/reconversion pathway, including phase transformation, intermediate formation, nucleation or reconstruction barriers at metal–LiX interfaces, and Li transport or trapping within converted nanocomposite domains [19, 20, 64, 65]. These processes should be invoked only when supported by phase‐, intermediate‐, or local‐structure‐sensitive evidence rather than by voltage‐profile shifts alone.

#### 2.2.6 Electrode‐Level Kinetics

Electrode‐level kinetics refer to the apparent electrochemical response governed by composite‐electrode architecture and testing conditions, including ionic transport through the pore network, electronic percolation through the carbon‐binder domain (CBD), particle/contact integrity, wetting/accessibility, interphase or contact resistance, electrode density/thickness, and formation history [66, 67, 68, 69]. Therefore, rate capability, polarization, or hysteresis changes measured in porous electrodes should not be assigned directly to intrinsic material‐level kinetics unless electrode‐level contributions are separately controlled or diagnosed.

**FIGURE 2:**
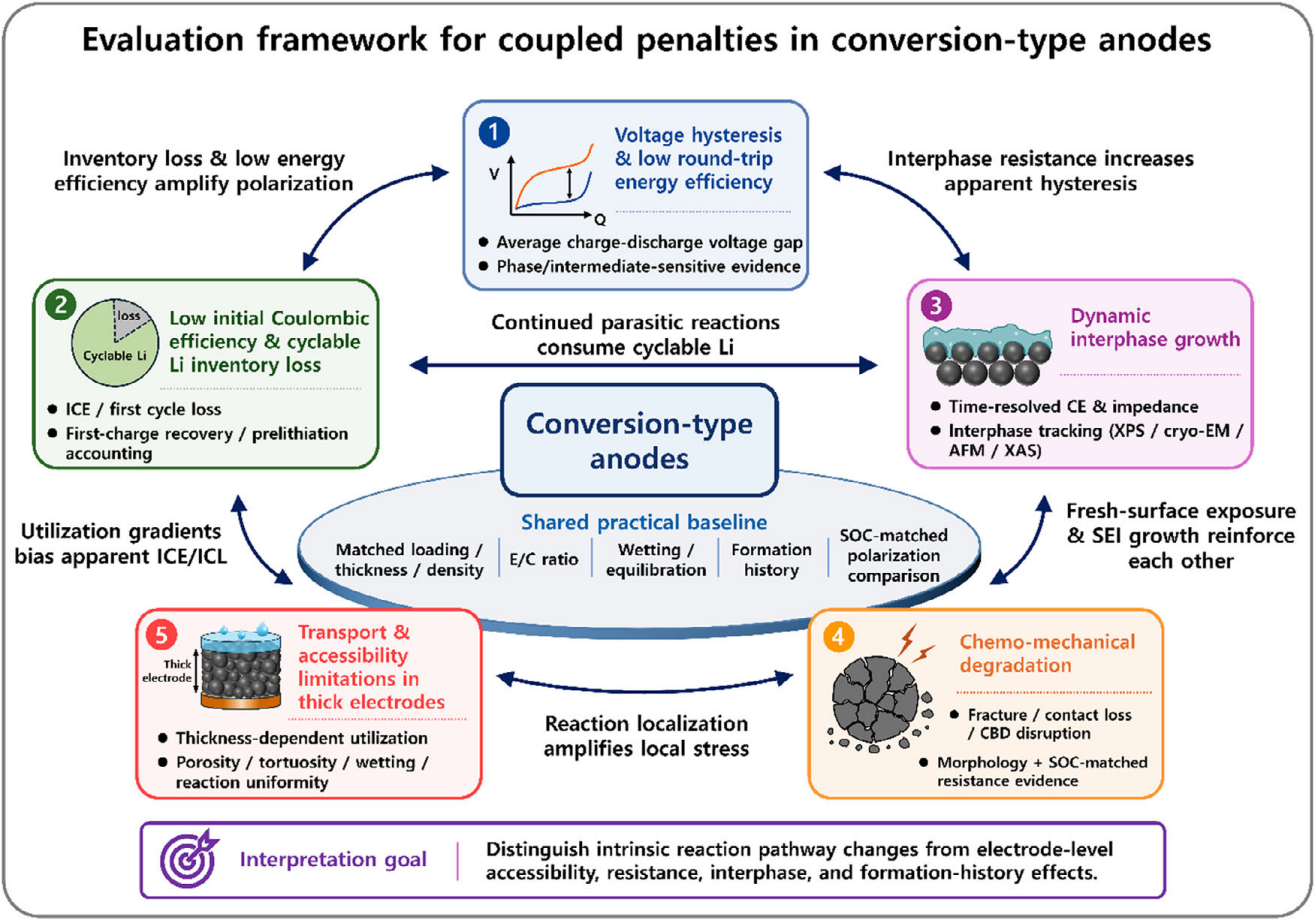
Evaluation framework for coupled penalties in conversion‐type anodes under practical cell conditions.

Taken together, these working definitions establish the interpretive frame used throughout this review. As summarized in Figure 2, comparisons of conversion‐type anodes should first be anchored to a shared practical baseline, including matched loading/thickness/density, electrolyte amount or E/C, wetting/equilibration state, formation history, and resistance or polarization evaluated at matched SOC [24, 40, 63, 70]. This baseline is required because changes in voltage hysteresis, ICE, interphase stability, mechanical integrity, or rate capability can arise from electrode‐level accessibility and resistance effects rather than from intrinsic changes in the conversion/reconversion pathway. Figure 2 also links each penalty to representative diagnostic checks, emphasizing that single‐observable claims should be avoided unless the corresponding electrochemical, structural, interfacial, and transport evidence is considered together. Accordingly, the validation checklists in the following sections are organized to emphasize penalty‐specific diagnostics under this shared baseline, rather than to restate the same general comparison criteria in each section.

The schematic summarizes five recurring penalties: voltage hysteresis and low round‐trip energy efficiency, low ICE and cyclable‐Li inventory loss, dynamic interphase evolution, chemo‐mechanical degradation, and thick‐electrode transport/accessibility limitations. The shared practical baseline and representative coupling pathways indicate how these penalties can amplify, mask, or redistribute one another. The listed diagnostics indicate the minimum evidence needed to distinguish intrinsic conversion/reconversion pathway changes from electrode‐level accessibility, resistance, interphase, and formation‐history effects.

### 2.3 Coupling Premise and Why a Single‐Observable Interpretation Can Mislead

Following the working definitions and shared practical baseline above, a central premise of this review is that the major penalties in conversion‐type electrodes are coupled and frequently co‐evolve; therefore, progress inferred from a single observable can misidentify the dominant penalty and lead to mechanistic misattribution [15, 71]. For example, lower observed polarization or narrower measured voltage hysteresis does not necessarily indicate a reduced intrinsic conversion barrier, because similar trends can arise from incomplete conversion, altered reconversion pathways [19, 20, 21, 72], or from electrode polarization and utilization limits in porous composite electrodes when pore ionic resistance becomes dominant [18, 70, 73]. Conversely, a strategy that narrows the voltage hysteresis can still increase irreversible lithium consumption if it increases reactive surface area or accelerates interphase formation; lower observed polarization should therefore not be interpreted directly as improved intrinsic kinetics without jointly examining lithium‐inventory loss and resistance evolution [37, 71].

Constraints arising in composite electrodes add another layer of coupling. Electrolyte amount and wetting state strongly influence how uniformly the electrode becomes accessible and can bias the spatial distribution of reaction and resistance growth, thereby shaping impedance rise and utilization nonuniformity during cycling [70, 74]. Formation conditions can likewise shape early interphase chemistry and the subsequent impedance response by altering the relative contributions of interphase‐related resistance, pore ionic‐transport resistance, and charge‐transfer resistance [37, 38]. Results obtained under electrolyte‐excess or lightly loaded conditions may not translate to thick, high‐areal‐loading composite electrodes.

Accordingly, the five penalties discussed below are treated as coupled rather than isolated outcomes. Mechanistic interpretation throughout this review follows a matched‐comparison framework that considers electrode geometry, electrolyte amount, and wetting state, formation history, and resistance or impedance evaluated at matched SOC.

### 2.4 Cross‐Coupling Logic of the Five Penalties

Although the five penalties are discussed in separate sections for clarity, they should not be interpreted as independent failure modes. In practical composite electrodes, each penalty can amplify, mask, or redistribute another, making single‐observable interpretation unreliable. As summarized in Figure 2, measured voltage hysteresis is coupled to electrode‐scale polarization and interphase/contact resistance; therefore, a changed voltage gap should be interpreted together with round‐trip energy efficiency, SOC‐matched resistance, and phase/intermediate evidence when pathway changes are claimed [19, 20, 39, 66]. Chemo‐mechanical degradation and dynamic interphase growth are likewise mutually reinforcing: fracture and contact loss expose fresh reactive surfaces, while interphase/internal passivation increases resistance and reaction localization, further accelerating mechanical damage [4, 39, 75]. In thick electrodes, ionic gradients, incomplete wetting, and heterogeneous current pathways can localize conversion reactions, increasing local overpotential, stress, underutilization, and contact degradation [26, 27, 28]. Utilization gradients or evolving wetting/accessibility can also bias apparent ICE/first‐cycle irreversible capacity loss (ICL) interpretation by changing the electrochemically active fraction and first‐cycle recovery range; therefore, ICE comparisons should be made under matched wetting, electrolyte amount, and formation conditions [17, 40, 69, 76, 77]. Finally, mitigation strategies can create trade‐offs: nanostructuring can reduce polarization but increase surface‐driven lithium consumption [39, 64, 78], while prelithiation can recover deliverable capacity without necessarily suppressing continuing parasitic reactions [36, 79]. These coupling pathways are the basis for the penalty‐specific diagnostic checks used throughout this review.

## 3 Voltage Hysteresis and Low Round‐Trip Energy Efficiency

Voltage hysteresis is a central practical limitation of conversion‐type anodes because it directly penalizes round‐trip energy efficiency even when high gravimetric capacity is retained [78, 80]. In practice, measured voltage hysteresis is a combined outcome of intrinsic material‐level kinetics [19, 21], and electrode‐level kinetic or polarization contributions, including porous‐electrode transport [66], and incomplete wetting/accessibility in porous composite electrodes [17, 63]. Accordingly, a change in the measured voltage gap should not be assigned to intrinsic hysteresis mitigation unless electrode‐level polarization, wetting, and resistance contributions are controlled or separately diagnosed.

### 3.1 Origins and Representative Hypotheses

#### 3.1.1 Pathway Asymmetry between Discharge and Charge

A widely accepted origin of large voltage hysteresis in oxide conversion anodes is discharge/charge pathway asymmetry: lithiation (conversion) and delithiation (reconversion) proceed via different intermediate phases and reaction sequences, even when the overall reaction is nominally reversible [19, 21].

**FIGURE 3:**
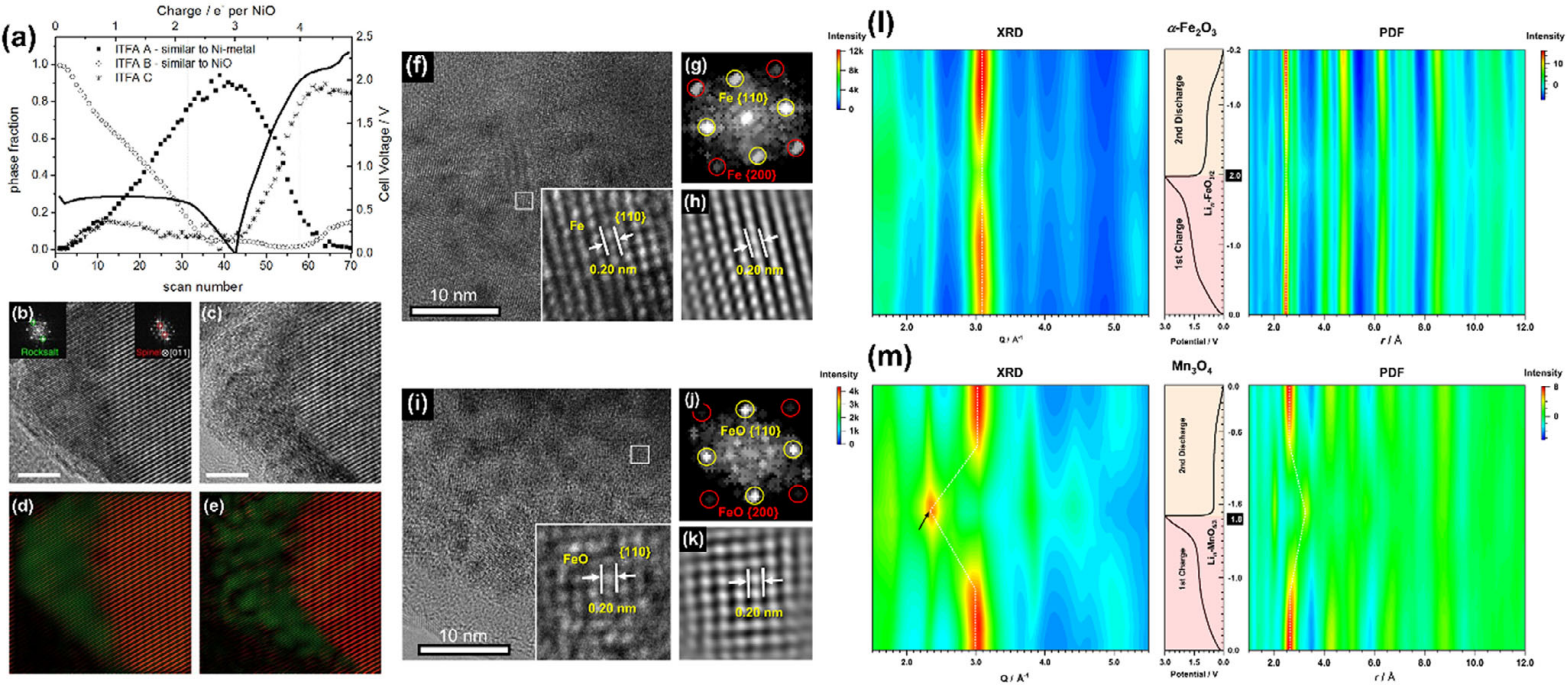
Multi‐scale evidence for voltage hysteresis in conversion‐type oxide anodes. (a) *Operando* voltage profile of NiO during the first cycle and cycle‐dependent phase‐fraction evolution, showing that discharge and charge populate different intermediate target‐factor‐analysis components and therefore follow asymmetric reaction pathways. Reproduced with permission [19]. Copyright 2014, Springer Nature. (b–e) In situ high‐resolution TEM (HRTEM) and phase‐filtered images of Fe_3_O_4_ during early lithiation, resolving sharp spinel/rocksalt phase boundaries and nonuniform phase‐front propagation within a single particle rather than a spatially homogeneous bulk transformation. Adapted with permission [81]. Copyright 2016, Springer Nature. (f–m) Charged‐state microscopy/fast Fourier transform and total‐scattering X‐ray diffraction (XRD)/pair distribution function analyses of Fe‐ and Mn‐oxide systems, showing that reconversion can proceed through non‐equilibrium oxide products, including bcc‐FeO and zincblende‐MnO, rather than simply regenerating the expected equilibrium monoxide. Adapted with permission [20]. Copyright 2021, Springer Nature.

Boesenberg et al. [19] reported, by operando phase‐fraction analysis, that NiO populates different intermediate fractions during discharge and charge (Figure 3a), indicating that the voltage gap is tied to pathway asymmetry rather than a simple ohmic offset. In situ X‐ray absorption near edge spectroscopy work by Jang et al. [82] provides complementary NiO‐specific spectroscopic support, showing that a single, simple reversible pathway does not capture the electronic‐structure evolution during charge and discharge. Comparable path asymmetry has also been reported in MnO/Mn_3_O_4_, CoFe_2_O_4_, Co_3_O_4_, CuS, MoS_2_, and CoP [45, 46, 47, 83, 84, 85, 86]. Non‐oxide systems such as Li–CoS_2_ [48] and FeP [49] also indicate that the same logic extends beyond oxides.

Cabana et al. [1] and Yao et al. [87] provide the mechanistic basis for this interpretation: reconversion must rebuild MX from a highly dispersed metal–Li_2_O matrix, so the charge step is governed by interfacial reconstruction in an electronically insulating environment. Consistent with that picture, Hua et al. [20] showed from charged‐state microscopy and total‐scattering analysis that Fe‐ and Mn‐oxide systems can yield non‐native oxide products such as body‐centered‐cubic FeO and zincblende MnO (Figure 3f–m), rather than simply returning to the expected equilibrium monoxide. Mechanical stress can further bias local pathway selection and contribute to the observed hysteresis [88].

#### 3.1.2 Interphase and Electrode‐Scale Heterogeneity: Insulating LiX Matrices, Reaction Localization, and Voltage Shifts Arising from Porous‐Electrode Effects

Voltage hysteresis can also be amplified—or partly masked—by interphase evolution and heterogeneity in porous composite electrodes. He et al. [81] visualized by in situ TEM that lithiation in Fe_3_O_4_ proceeds through sequential intercalation and conversion with distinct phase fronts and local phase coexistence within a single particle (Figure 3b–e), showing that the measured voltage profile can already contain spatially nonuniform reaction progress before electrode‐thickness effects are added. Cabana et al. [1] had already emphasized that insulating LiX products and metal–LiX nanocomposites favor heterogeneous reaction propagation, and porous‐electrode transport limitations then add thickness‐dependent polarization to the same signal [66]. Correlative *operando* microscopy further showed that heterogeneous conversion in Fe_3_O_4_ composite electrodes can produce migrating reaction fronts and persistent under‐reacted/over‐reacted regions, even when the intrinsic conversion energetics remain unchanged [26, 89]. A closely related NiO study by Lin et al. [21] is also important in this context. By combining spectroscopy with three‐dimensional imaging across multiple charge states, they showed that lithiation/delithiation in NiO nanosheets can proceed via spatially dependent phase evolution rather than a single, spatially uniform pathway, reinforcing the idea that measured hysteresis can reflect heterogeneous reaction progress in addition to pathway asymmetry.

Accordingly, changes in electrode microstructure—such as CBD connectivity [67, 68] or porosity/tortuosity distribution [69]—can reduce electrode polarization and narrow the measured voltage hysteresis without necessarily changing the intrinsic conversion barrier.

Electrolyte wetting and pore filling can further reshape the measured voltage profile by altering the fraction of the electrode that is electrochemically active. When wetting is incomplete or evolves with time [17, 63], the region of the electrode that actually participates in the reaction can shift across the thickness during cycling, causing changes in the measured voltage hysteresis [90].

Therefore, voltage hysteresis narrowing alone should not be taken as evidence of pathway convergence unless improvements in reaction uniformity across electrode thickness, reaction distribution, and electrode utilization are demonstrated under a matched electrode architecture and matched electrolyte amount/wetting state [66, 69, 81].

#### 3.1.3 Chemo‐Mechanical and Interphase Evolution Amplify Polarization and Hysteresis

Conversion anodes undergo large volume changes and repeated interfacial reconstruction during conversion–reconversion, which can drive particle cracking/fragmentation and progressive loss of electronic contact [75, 91]. Beyond active‐material fracture, the changes in CBD connectivity and particle–particle contact can reshape the electronic percolation backbone of the composite electrode, thereby increasing polarization and broadening voltage dispersion even without a change in intrinsic conversion energetics [92, 93].

Because crack‐driven surface renewal exposes fresh reactive interfaces, continued electrolyte reduction and solid electrolyte interphase (SEI) thickening can persist well beyond the first cycle [37, 39]. In magnetite (Fe_3_O_4_) conversion electrodes, electrochemical and surface analyses show that SEI formation and its continued evolution can contribute to kinetic degradation and increasing polarization, underscoring that voltage hysteresis drift may reflect evolving interphase/contact resistance rather than convergence of the conversion/reconversion pathway [94, 95, 96].

These effects are further amplified under thick‐electrode or lean‐electrolyte conditions, where localized reaction fronts couple to ionic transport gradients and current constriction, accelerating both impedance rise and hysteresis evolution [17, 63, 97].

### 3.2 Mitigation Strategies

#### 3.2.1 Pathway And Interface Engineering toward Reduced Hysteresis

This class of strategies aims to reduce hysteresis by lowering reconstruction penalties at metal–LiX interfaces and favoring more reversible conversion/reconversion pathways, rather than merely reducing external polarization [80]. Recent literature also suggests that a credible hysteresis‐mitigation claim should coincide with altered metastable intermediate persistence or modified reaction‐front propagation, rather than with a shifted galvanostatic profile alone; this point has been emphasized in both direct Mn_3_O_4_ hysteresis studies and broader in situ TEM analyses of conversion‐type electrodes [98, 99]. In oxide conversion anodes, in situ studies have shown that framework‐retaining or topotactic/displacement‐like reconversion pathways are associated with altered intermediate evolution at the phase level [20, 21, 72]. Accordingly, credible hysteresis mitigation should not be inferred solely from a shifted voltage profile, because similar shifts can also arise from improved percolation or wetting [63].

In this context, the charged‐state analyses of Hua et al. [20] provide a reference case for pathway‐based mitigation claims: if a strategy truly reduces hysteresis by altering the conversion/reconversion pathway, it should modify the charged‐state phase selection or reconversion product rather than merely shift the voltage profile through improved wetting, percolation, or reduced electrode‐scale resistance [63].

#### 3.2.2 Nanostructuring and Nanoconfinement

Particle downsizing and nanoconfinement can mitigate polarization by shortening transport lengths and preserving electronic contact during large‐volume‐change reactions [1, 78, 100]. Representative examples include yolk‐shell NiO powders [58], in situ carbon‐coated yolk‐shell V_2_O_3_ microspheres [59], and multilayer CuO@NiO hollow spheres [60], which buffer volume change while shortening transport lengths and maintaining electronic continuity during cycling. Similar logic has been applied to hollow Co_3_O_4_, low‐dimensional CuS and MoS_2_, morphology‐controlled WO_3_ and WS_2_ nanocrystals, Co_2_P quantum dots, and one‐dimensional chalcogenides, where nanoscale confinement or morphology control simultaneously improves transport, structural accommodation, and reaction evolution [45, 46, 50, 51, 52, 61]. As summarized in Table 1, many of these nanostructured examples are best interpreted as proof‐of‐concept demonstrations of nanoscale confinement, transport shortening, and strain accommodation unless practical descriptors such as loading/thickness/density, E/C ratio, wetting/equilibration, and full‐cell context are reported. However, because nanostructuring also increases interfacial area and electrolyte demand, the narrowing of measured voltage hysteresis should not be interpreted as intrinsic hysteresis mitigation unless interphase growth is controlled in parallel [37, 39].

#### 3.2.3 Conductive Frameworks and Electrode Architecture

Beyond particle downsizing, conductive frameworks and electrode architectures can reduce polarization in porous composite electrodes by keeping conversion domains electronically wired and suppressing local current constriction [27, 66]. Fe_3_O_4_‐based Cu nano‐architectures, Fe_3_O_4_@Fe_3_C–C yolk–shell structures, and Co_2_P quantum‐dot/carbon hybrids all follow this principle by stabilizing transport pathways during repeated conversion and reconversion [50, 53, 54]. Carbon‐coated Ni‐Mn‐Co‐O inverse opals [55], mesoporous NiO nanosheets [57] grown on carbon cloth, and CoS2 nanoparticle/graphene/CNT aerogels [56] all follow this principle by maintaining continuous electronic pathways and suppressing local current constriction during cycling. More broadly, carbon‐based oxide and sulfide nanocomposites combine short diffusion length with sustained percolation, but their success should be judged by utilization uniformity and resistance evolution rather than by capacity alone [8, 101]. Because CBD morphology and the dual‐porosity electrode architecture strongly influence effective ionic/electronic pathways, architecture‐based improvements should be evaluated by assessing utilization uniformity across the electrode thickness and impedance/polarization trajectories, rather than solely by voltage profile shifts [67, 68, 69].

#### 3.2.4 Electrolyte Design, Wetting, and Formation Protocols

Electrolyte design, wetting, and formation protocols can indirectly reduce measured hysteresis by lowering interphase‐related resistance growth and making the reaction more uniform, but they should not be taken as evidence of a lower intrinsic conversion barrier unless phase‐sensitive measurements also show a real pathway change [37, 38, 39, 40]. Because CBD morphology and pore‐network tortuosity/connectivity strongly influence effective ionic/electronic pathways, architecture‐based improvements should be evaluated by assessing utilization uniformity across the electrode thickness and by examining impedance/polarization trajectories rather than solely by voltage‐profile shifts [63, 70]. Under practical composite‐electrode conditions, improvements induced by electrolyte design or formation are therefore better interpreted as hysteresis mitigation arising from improved accessibility or slower resistance growth, unless *operando* phase evidence also supports a genuine pathway change [25, 38, 39, 40].

### 3.3 Integrated Interpretation and Validation Checklist

#### 3.3.1 Integrated Interpretation

Overall, voltage hysteresis in conversion anodes should be interpreted as the combined result of asymmetric conversion/reconversion pathways and polarization in porous composite electrodes [66], not as a property of the active material alone. Because SEI growth and contact/percolation evolution can shift the resistance partition with cycling [39], voltage‐hysteresis trends should therefore be interpreted together with round‐trip energy efficiency [80] and resistance or impedance evaluated at matched SOC under a transparent protocol [25]. Claims about pathway changes should be reserved for cases where phase‐ or intermediate‐sensitive evidence is available, ideally from *operando* methods that resolve structural evolution during cycling [20, 86, 102, 103].

The following scenarios are representative diagnostic patterns, not an exhaustive classification of all possible outcomes. They are distinguished by the co‐evolution of voltage hysteresis or round‐trip energy efficiency, resistance or impedance evolution, and ICE/CE behavior. When these observables change simultaneously, the result should be treated as a coupled response rather than forced into a single failure category.

Representative diagnostic scenario 1. Voltage hysteresis narrows, but round‐trip energy efficiency does not improve.

This pattern is more consistent with redistributed reaction utilization or electrode‐scale polarization than with confirmed pathway convergence. Voltage hysteresis can narrow when the reaction shifts within the voltage profile or when transport losses are redistributed, even if the intrinsic conversion barrier remains unchanged [66, 69]. If round‐trip energy efficiency does not improve, a smaller voltage gap should not be assigned to genuine hysteresis mitigation [80].

Representative diagnostic scenario 2. Early hysteresis reduction decays while impedance or resistance rises.

This pattern indicates that the initial improvement may be offset by resistance‐partition drift, interphase growth, contact loss, or conductive‐network degradation during cycling [25, 39]. In such cases, early voltage‐profile improvement should be interpreted together with SOC‐matched resistance evolution rather than as a stable change in the conversion pathway.

Representative diagnostic scenario 3. Voltage hysteresis decreases, but ICE worsens or early CE drift occurs.

This pattern should be treated as an efficiency trade‐off: reduced apparent polarization can coexist with increased lithium‐inventory loss or sustained parasitic reduction. Hysteresis mitigation should therefore be reserved for cases where round‐trip energy efficiency and CE improve together [39, 80, 104].

#### 3.3.2 Validation Checklist

1. Define how voltage hysteresis is quantitatively extracted: Report the hysteresis metric used, such as the average charge–discharge voltage gap over a defined capacity window, the capacity‐matched voltage difference between lithiation and delithiation, or an energy‐based hysteresis measure derived from the integrated voltage profiles. When peak separation from dQ/dV or cyclic voltammetry is used, scan rate and polarization effects should be explicitly discussed [105, 106].
2. Make round‐trip energy efficiency the primary outcome, not voltage hysteresis alone: A narrower voltage hysteresis does not necessarily translate into improved round‐trip energy efficiency, because energy loss depends on the integrated voltage gap over capacity. In thick or heterogeneous composite electrodes, a similar narrowing can also result from a shifted reaction range or from changes in ionic access and transport, rather than from a true reduction in the intrinsic conversion barrier [66, 69]. Therefore, voltage hysteresis trends should be interpreted in conjunction with thickness‐dependent polarization and uniformity of utilization [105, 106].
3. Use SOC‐matched resistance tracking to interpret voltage hysteresis evolution: Track impedance at comparable SOC with a consistent relaxation/measurement protocol, since SOC‐dependent impedance can masquerade as drift or improvement [25]. When impedance is not available, compare DC‐IR, overpotential, or polarization measured under equivalent conditions. Resistance‐partition trends help discriminate pathway effects from interphase/contact‐driven polarization.
4. Require phase/intermediate evidence for claims about pathway changes: To claim a change in conversion–reconversion pathway (e.g., altered intermediate population, reduced reconstruction penalty), include *operando* phase/intermediate tracking that directly supports the asserted mechanism, ideally using one or more of the following evidentiary levels: phase‐fraction evolution, metastable intermediate identification, or direct particle‐scale phase‐front visualization [19, 81, 86, 102, 103]. Otherwise, keep conclusions at the electrode‐response level [20].
5. Include at least one heterogeneity‐sensitive check: Provide a diagnostic that distinguishes improved utilization uniformity from a shifted electrochemically utilized volume (e.g., thickness dependence, a reaction‐distribution indicator, or controlled wetting/access tests) [69]. This matters because distribution losses can change voltage hysteresis without improving the intrinsic conversion barrier [63].

## 4 Low Initial Coulombic Efficiency and Cyclable‐Lithium Inventory Loss

Low ICE of conversion‐type anodes directly reduces the cyclable lithium inventory, thereby lowering the deliverable full‐cell energy, even when the half‐cell specific capacity appears high [36, 62]. Because ICLmust be compensated by positive‐electrode capacity oversizing, prelithiation, or other lithium sources, ICE should be treated as an inventory and energy‐density penalty in full cells.

For conversion‐type anodes, achieving practically sufficient ICE for realistic full‐cell balancing remains challenging because the measured ICE reflects the coupled contributions of (i) irreversible lithium consumption by electrolyte reduction and SEI formation [37, 39], (ii) conversion‐induced creation of fresh interfaces/reactive surface area associated with metal–LiX nanocomposite formation [64], and (iii) incomplete reconversion and lithium trapping that limits first‐charge recovery [64, 65]. A broader caution comes from systematic first‐discharge studies of iron‐ and manganese‐oxide anodes, which show that the initial lithiation can contain extra capacity beyond nominal conversion. Therefore, the first‐cycle discharge capacity should not be equated with recoverable cyclable lithium without independent phase or electrolyte diagnostics [107].

Because measured ICE is highly sensitive to cell condition (areal capacity/loading, porosity/tortuosity, negative‐to‐positive capacity (N/P) ratio), electrolyte availability and wetting, and the complete formation trajectory (currents, temperature, rests/holds, and cutoff logic), ICE should only be compared under explicitly reported electrolyte conditions (e.g., electrolyte amount or E/C) with fully specified wetting/equilibration and formation protocols [40, 76, 77].

### 4.1 Origins and Representative Hypotheses

#### 4.1.1 First‐Cycle Irreversible Capacity From Electrolyte Decomposition and SEI Formation

**FIGURE 4:**
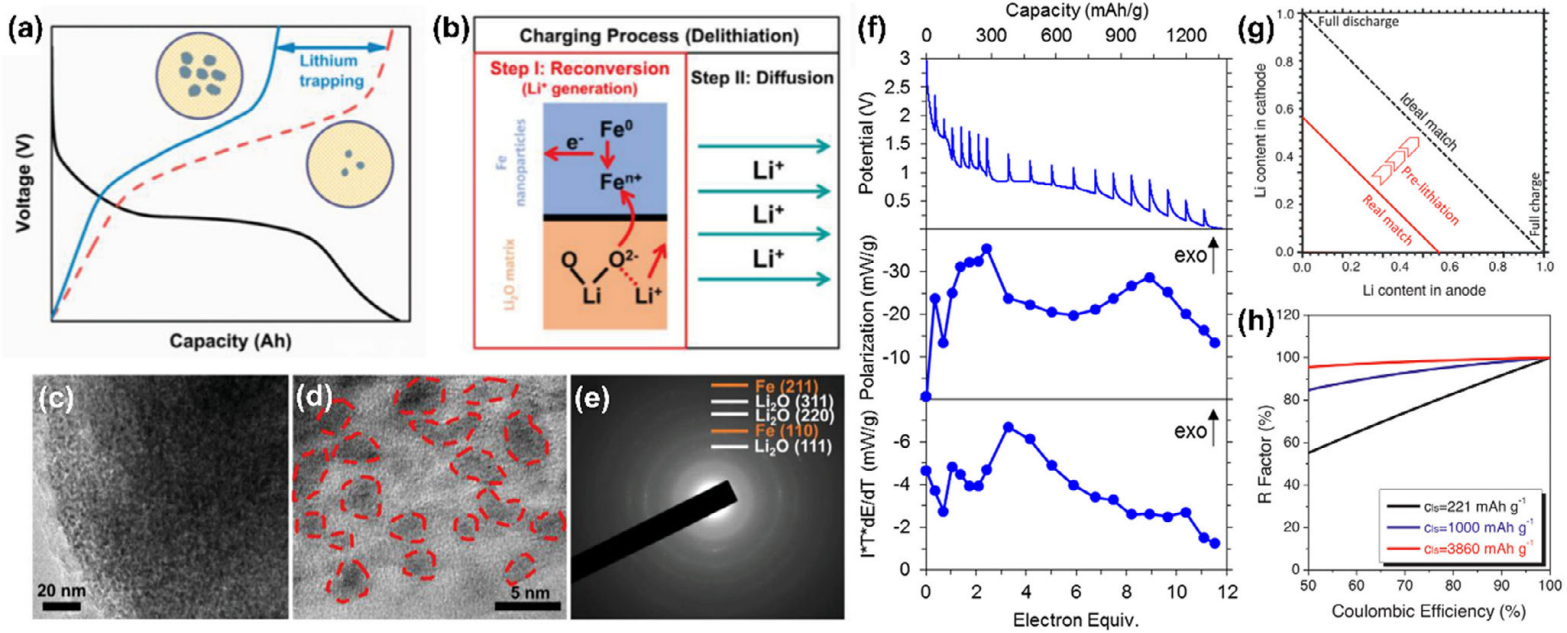
Mechanistic and full‐cell interpretations of low ICE in conversion‐type anodes. (a,b) Schematic representation of lithium trapping‐induced low ICE and the two‐step first delithiation process in Fe‐based conversion anodes, where reconversion first generates Li^+^ and subsequent Li^+^ diffusion out of the Li_2_O‐rich matrix can become rate‐limiting. (c–e) Discharged‐state high‐resolution transmission electron microscopy (HRTEM and selected‐area electron diffraction (SAED) evidence showing Fe nanoparticles dispersed in a Li_2_O matrix, i.e., the nanostructural starting point from which sluggish solid‐state reconversion can leave Li trapped on the subsequent charge. (a–e) Adapted with permission [65]. Copyright 2023, Wiley. (f) Potential and calorimetric analysis of Fe_3_O_4_ first lithiation, showing extra heat output below ≈0.86 V that is consistent with electrolyte reaction and associated SEI formation beyond nominal oxide reduction. Adapted with permission [108]. Copyright 2018, American Chemical Society. (g) Full‐cell lithium‐matching diagram showing that low anode CE shifts the practical matching line away from the ideal case and creates a lithium‐inventory deficit that can be compensated by prelithiation. (h) Reduction‐factor analysis as a function of anode CE for different lithium‐source capacities, showing that the benefit of prelithiation depends on both anode CE and the amount of compensating lithium supplied by the lithium source. (g,h) Adapted with permission [36]. Copyright 2021, Wiley.

Electrolyte reduction and SEI formation irreversibly consume cyclable lithium during the anode's initial low‐potential exposure. The extent of this loss depends on electrolyte composition and formation conditions [37], time at low potential and temperature [109], and reactive surface area [39, 64]. In conversion‐type anodes, the first conversion generates a highly dispersed metal–LiX nanocomposite and extensive new interfaces [64], which intensify early parasitic reactions and make ICL especially sensitive to formation conditions. Accordingly, ICE/ICL should be evaluated together with at least one early‐life resistance or impedance measurement, rather than solely by capacity, because interphase growth and contact evolution directly affect polarization and the recoverable capacity on first charge [40, 109]. Huie et al. [108] combined isothermal microcalorimetry with *operando* X‐ray absorption spectroscopy during Fe_3_O_4_ lithiation and identified additional heat output below ≈0.86 V (Figure 4f) beyond oxide‐reduction‐only behavior, consistent with electrolyte reaction and associated SEI formation. That calorimetric signal is consistent with parasitic lithium consumption during the first cycle. More specifically, *operando* and post‐mortem studies on Fe_3_O_4_ show that the first‐cycle irreversible loss arises from interphase formation and parasitic electrolyte reduction on fresh surfaces generated during conversion [94, 110].

#### 4.1.2 Lithium Trapping and Incomplete First‐Charge Recovery after Conversion

In conversion‐type anodes, the first delithiation must reconvert the metal–LiX nanocomposite toward MX or related phases. When reconversion is incomplete or ion/electron transport is locally blocked, Li can remain trapped as LiX in electronically or ionically isolated regions, thereby lowering first‐charge recovery and ICE [64, 65]. Cao et al. [65] proposed a two‐step picture for the first delithiation of Fe‐based conversion products (Figure 4a,b), in which reconversion first generates mobile Li^+^ and is then followed by Li^+^ diffusion out of the Li_2_O‐rich matrix; when the reconversion step is sluggish, lithium trapping persists, and the charge trajectory deviates from the fully recoverable case. Their discharged‐state high‐resolution transmission electron microscopy (HRTEM) images and selected‐area electron diffraction (SAED) data (Figure 4c–e) show Fe nanoparticles embedded in a Li_2_O‐rich matrix, which gives this interpretation direct physical support. Phase‐/intermediate‐sensitive studies on cobalt oxides [111], MoS_2_ [46], and NiO [19] support the same broader conclusion that first‐charge irreversibility can persist through residual converted domains or incomplete reconversion. A conceptually related outcome has also been reported for the conversion‐alloying Zn_2_SnO_4_ anode, where incomplete lithium release and matrix reactivity generate a distinct form of trapping/recovery limitation [112].

Phase‐ and intermediate‐sensitive measurements directly reveal this first‐charge irreversibility. For example, in situ/*operando* X‐ray absorption spectroscopy (XAS) on cobalt oxides shows residual metallic Co that persists on charge due to phase isolation [111]; *operando*/*ex situ* X‐ray diffraction (XRD)/XAS analyses on MoS_2_ indicate that the 2H → 1T‐Li_x_MoS_2_ transformation is only partially reversible upon charge [46]; and asymmetric conversion–reconversion with residual converted domains has likewise been reported in NiO [19]. Cao et al. [65] further showed that, after lithiation, Fe nanoparticles remain embedded in a Li_2_O‐rich matrix in the discharged state, providing the physical starting point from which sluggish solid‐state reconversion can leave Li trapped on the first charge.

At the electrode scale, trapping can be amplified by unstable electronic percolation and evolving CBD networks during large phase/volume changes [68, 113], as well as by pore‐network limitations that restrict ionic access to parts of the composite [69]. Accordingly, an ICE gain should not be viewed as reduced parasitic consumption unless first‐cycle profiles show higher first‐charge recovery without a compensating rise in polarization or early resistance under identical cutoffs and constraints [38, 40].

#### 4.1.3 Protocol and Cell‐Context Dependence: Wetting/Availability, Formation Trajectory, and Cutoff Logic

ICE is strongly influenced by electrolyte wetting and availability, as these factors determine the extent of electrochemically accessible electrode surface during the first cycle, especially under lean electrolyte conditions relevant to cell manufacturing [17, 76]. ICE also depends on formation protocol variables such as current, temperature, rest/hold steps, and cutoff logic [77, 109]. Because formation can shift the electrochemically utilized capacity range and modify early resistance/impedance response [40], ICE comparisons should use matched wetting/equilibration procedures and identical cutoff logic, and should report first‐cycle voltage profiles together with at least one early‐life resistance indicator to distinguish true chemistry‐driven gains from accessibility‐driven shifts [38, 40]. Accordingly, when formation or cutoff conditions are presented as strategies for improving ICE, first‐cycle coulometry should be interpreted alongside diagnostic measurements and SOC‐resolved impedance to distinguish a real reduction in parasitic loss from a nominal gain caused by a shifted recovery range [114, 115].

### 4.2 Mitigation Strategies

A practical strategy for mitigating low ICE and first‐cycle irreversible capacity loss should first identify which component of the lithium loss is being addressed. In general, three routes are possible. First, parasitic lithium consumption can be reduced by suppressing electrolyte reduction and promoting a more passivating interphase through electrolyte, coating, and formation‐protocol design [37, 38, 39, 40]. Second, first‐charge recovery can be improved by reducing reconversion limitations or lithium trapping, which requires preserving ionic accessibility and electronic percolation through the converted metal–LiX matrix [64, 65]. Third, the remaining cyclable‐lithium deficit can be compensated by external lithium sources, such as prelithiation or sacrificial lithium additives [33, 34, 35, 36]. These routes should not be conflated: the first two aim to reduce anode‐side irreversible loss mechanisms, whereas prelithiation primarily restores lithium inventory at the full‐cell level without necessarily improving intrinsic anode reversibility.

#### 4.2.1 Lithium Inventory Management: Prelithiation and Sacrificial Lithium Sources

Prelithiation and sacrificial lithium sources compensate for first‐cycle lithium loss by supplying additional active lithium, recovering full‐cell deliverable capacity even when anode ICE remains limited [36, 62]. Recent practical reviews emphasize that such methods should be judged not only by nominal capacity recovery, but also by the amount of delivered lithium, process compatibility, air stability, and manufacturability [33, 34, 35]. Representative approaches include cathode/anode prelithiation, prelithiation using stabilized lithium metal powder, Li‐rich alloy sources, and protected Li‐containing reagents [79, 116, 117, 118, 119, 120]. Because this method primarily manages lithium inventory, the supplied or delivered lithium should be reported in areal‐capacity units, such as mAh cm^−2^, and compared with the first‐cycle irreversible capacity of the target anode. For an anode with a first‐discharge areal capacity *Q*_dis,1_ and ICE = *Q*_ch,1_/*Q*_dis,1_, the anode irreversible capacity is *Q*_ICL_ = *Q*_dis,1_ – *Q*_ch,1_ = *Q*_dis,1_(1‐ICE). Practical prelithiation studies should therefore specify whether the supplied lithium is below, approximately equal to, or above this irreversible‐capacity requirement. Under‐supply leaves a remaining lithium‐inventory deficit, whereas excessive supply can distort full‐cell balancing or leave residual active lithium. Therefore, the supplied lithium amount should be evaluated under the same electrolyte amount, wetting, and formation protocol used in the target cell design [40, 76, 77].

At the full‐cell scale, Jin et al. [36] formalized the same problem as a lithium‐inventory deficit that can be compensated by prelithiation, and the matching logic summarized in Figure 4g,h makes clear that this intervention restores deliverable lithium inventory without demonstrating improved intrinsic reversibility of the anode [33, 34, 35, 36].

#### 4.2.2 Electrolyte and Formation‐Protocol Engineering to Raise Intrinsic ICE

Electrolyte design (salts/solvents/additives) and formation protocols can raise chemistry‐limited ICE by suppressing early parasitic reduction, promoting passivating interphases, and improving initial wetting and current distribution during the first lithiation [37, 38, 76]. However, the same formation variables can also change first‐charge recovery by changing the fraction of the electrode that becomes electrochemically accessible during the first charge and by altering the early impedance response, so protocol transparency is essential for interpretation [40, 77, 109]. A genuine chemistry‐driven improvement in ICE should therefore be claimed only when first‐charge recovery and/or early CE improve without a parallel increase in first‐charge polarization or early‐life resistance, under matched electrolyte amount, wetting/equilibration history, and cutoff conditions [40].

#### 4.2.3 Interfacial Stabilization via Surface Coatings and Artificial SEI

Surface coatings and artificial interphases are included here mainly as interfacial‐design precedents, because most of the established coating literature comes from graphite and Si‐based anodes rather than from conversion anodes [121, 122, 123, 124]. For conversion‐type anodes, the key translation requirement is that the coating remain conformal and ion‐permeable under repeated conversion strain; otherwise, delamination/cracking will reopen fresh surface and sustain first‐cycle loss or subsequent parasitic reactions [75]. Accordingly, coating strategies in conversion anodes should be framed as interphase‐durability approaches that can reduce first‐cycle parasitic loss and stabilize early resistance growth, rather than as direct solutions to lithium trapping unless first‐charge recovery also improves [38, 40, 124]. Coating efficacy should therefore be judged not only by ICE, but also by early‐life impedance/polarization and subsequent CE stabilization under matched practical constraints [38, 40].

#### 4.2.4 Mitigating Lithium Trapping: Preserving Ionic Accessibility and Electronic Percolation

Architectures that preserve ionic accessibility and continuous electronic pathways can reduce the apparent severity of lithium trapping by increasing the fraction of active material that participates reversibly. However, in conversion anodes, the same designs that improve electronic connectivity or pore accessibility can also increase interfacial area and aggravate SEI formation. The design goal is therefore not simply to maximize nanoscale mixing, but to maintain continuous electronic pathways while limiting excessive interfacial area so that reconversion can proceed throughout the electrode [101, 125, 126]. This requirement is especially critical in conversion‐type anode systems, where electronically insulating Li_2_O‐ or Li_2_S‐rich matrices can isolate active domains during the first conversion [19, 46, 111]. Accordingly, these strategies are most convincing when first‐cycle recovery improves without a parallel rise in polarization or early resistance [65, 77].

### 4.3 Integrated Interpretation and Validation Checklist

#### 4.3.1 Integrated Interpretation

Taken together, low ICE in conversion anodes should be attributed to parasitic lithium consumption during initial interphase formation and to incomplete first‐charge recovery caused by reconversion limitations or lithium trapping [62, 64, 65, 108]. Prelithiation addresses the resulting lithium‐inventory deficit at the full‐cell level but, by itself, does not demonstrate improved intrinsic ICE [33, 34, 35, 36]. Accordingly, numerical ICE gains should not be interpreted solely from coulometry. Similar improvements can arise from reduced parasitic losses, improved first‐charge recovery, or external lithium compensation.

Case 1. ICE increases and first‐charge recovery increases at fixed voltage limits.

Interpret the result as improved reconversion or accessibility, not necessarily as reduced parasitic chemistry. Before attributing the gain to intrinsic reversibility, residual converted domains and electrode‐network integrity should be checked [64, 65, 113].

Case 2. ICE improves, but early CE drifts downward, or resistance rises faster.

Treat the outcome as continued interphase growth rather than stabilized chemistry if early CE drifts or resistance rises; ICE alone is insufficient [25, 37, 38].

Case 3. Deliverable capacity is recovered using prelithiation or sacrificial lithium sources.

Treat the intervention as lithium‐inventory management rather than intrinsic ICE improvement, and verify that CE and resistance do not deteriorate after compensation [36, 116, 117].

#### 4.3.2 Validation Checklist

1. Define the full‐cell balancing context before interpreting ICE: Report the full‐cell balancing conditions (N/P ratio, areal capacities, cathode oversizing) and clarify whether ICE is discussed as half‐cell coulometry or as a full‐cell inventory penalty [36, 62, 127].
2. Interpret ICE together with first‐cycle discharge and first‐charge recovery: Provide first‐cycle charge/discharge profiles and first‐charge recovery so that changes in ICE can be interpreted in terms of parasitic lithium loss and incomplete first‐charge recovery [64, 65]. When kinetic limitation is central to the interpretation, galvanostatic intermittent titration technique measurements can provide complementary support for distinguishing nonequilibrium from quasi‐equilibrium behavior [128].
3. Require direct evidence for lithium trapping or reconversion limitation: If lithium trapping or reconversion limitation is part of the interpretation, include at least one diagnostic sensitive to residual LiX or phase evolution, such as *operando* phase‐sensitive measurements or equivalent quantitative evidence [64, 65]. Otherwise, keep the interpretation at the level of recovery limitation in composite electrodes.
4. Pair ICE with early CE and resistance evolution: Report early CE trends together with at least one early‐life resistance or impedance measurement to avoid labeling a shifted first‐cycle trajectory as reduced parasitic loss or improved first‐charge recovery [38]. Use matched‐SOC tracking when comparing resistance growth [25].
5. When prelithiation is used, quantify the amount of lithium supplied relative to the anode irreversible capacity and show that it does not conceal continued degradation: State the amount of lithium supplied by prelithiation in mAh cm^−2^, how the lithium is introduced, and at what stage prelithiation is applied. The supplied lithium should be compared with the anode first‐cycle irreversible capacity, *Q*_ICL_ = *Q*_dis,1_ – *Q*_ch,1_, under the same formation protocol and voltage limits used for the target cell. Indicate whether CE remains stable and resistance growth is suppressed after prelithiation, because additional lithium inventory can mask persistent parasitic reactions or interphase growth. When prelithiation is presented as a practical strategy, also discuss process compatibility, air stability, and manufacturability rather than merely reporting capacity recovery [33, 34, 35, 36].

## 5 Interphase Instability, Continuous Electrolyte Reduction, and Dissolution‐Mediated Cross‐Talk

Continuously evolving anode interphases are a dominant long‐term limiter in Li‐based cells because they sustain parasitic electrolyte reduction, leading to ongoing consumption of cyclable lithium and progressive increases in resistance during cycling [71, 129]. This perspective aligns with the classical SEI model and the foundational electrolyte literature, which attribute long‐term degradation to coupled lithium and electrolyte consumption and to impedance rise driven by continued reduction reactions [129, 130]. In conversion anodes, the issue is further exacerbated by repeated surface renewal induced by volume‐change‐driven fracture and by the high reactivity/catalytic activity of freshly formed metallic nanoparticles embedded in LiX matrices [39]. Consequently, the anode interphase often behaves not as a fully passivating static SEI but as a dynamic, composite interphase whose composition, impedance, and reactivity evolve with the conversion microstructure and local SOC [41, 131].

In this review, SEI evolution in working conversion anodes is therefore treated as a cycle‐ and SOC‐dependent process that should be connected to electrochemical consequences. Model interfacial experiments can identify elementary growth modes, but stabilization claims for working electrodes should be supported by cycling‐resolved CE, SOC‐matched resistance or impedance, and surface‐ or structure‐sensitive evidence rather than endpoint post‐mortem composition alone [42, 43, 94, 132].

### 5.1 Origins and Representative Hypotheses

#### 5.1.1 Persistent Electrolyte Reduction Driven by Repeated Fresh‐Surface Exposure and Electronically Leaky Interfaces

Cracking, pulverization, and loss/recovery of electronic contact can repeatedly rupture the SEI and expose fresh reactive surface [75], thereby re‐initiating electrolyte reduction beyond the initial formation step [39]. In conversion‐type anodes, repeated conversion–reconversion can further reorganize the electronic percolation network and buried metal–LiX interfaces [21], thereby prolonging reaction localization and local current constriction and sustaining parasitic reduction at the anode. Consistently, conversion electrodes often show a continuously evolving anode–electrolyte interphase during cycling rather than a fully passivated, static state [41]. At the working‐electrode level, this evolution should be distinguished from endpoint SEI composition. A stabilized SEI claim is more convincing when cycle‐resolved interphase evidence is linked to electrochemical signatures such as CE drift, SOC‐matched impedance or resistance growth, and polarization evolution. Operando atomic force microscopy, cryogenic‐electron microscopy, and XAS/X‐ray photoelectron spectroscopy (XPS) studies on conversion‐type electrodes show that interphase morphology and chemistry can evolve with cycle number, SOC, and electrolyte chemistry, so a single post‐mortem spectrum cannot by itself establish interphase stabilization [42, 43, 94].

**FIGURE 5:**
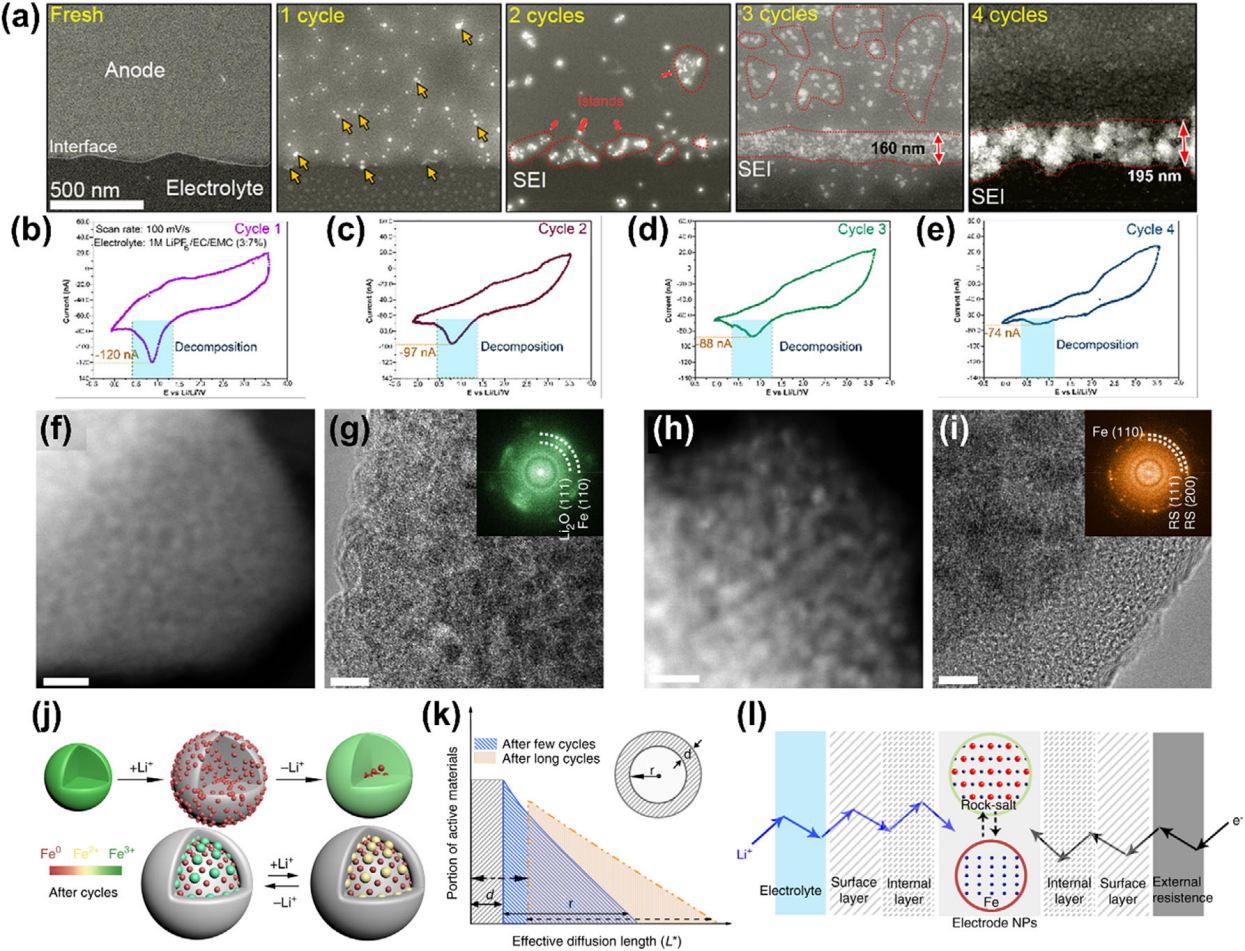
Dynamic interphase evolution and passivation‐layer growth in conversion electrodes. (a–e) *Operando* liquid‐cell STEM images and cycle‐resolved voltammograms of a model anode/electrolyte interface under a conventional LiPF_6_/ethylene carbonate‐ethyl methyl carbonate electrolyte, showing progression from a fresh interface to nanoparticle nucleation, island‐like growth, and a thicker, more continuous mosaic interphase over the first four cycles. Reproduced with permission [133]. Copyright 2023, American Chemical Society. (f–i) Later‐cycle microscopy of Fe_3_O_4_ conversion electrodes showing enlarged Fe‐containing bright regions and Li_2_O‐rich/passivation‐rich dark regions after prolonged cycling. (j–l) Schematic interpretation of later‐stage phase evolution, thickening of the effective diffusion length, and multilayer Li^+^/electron transport barriers imposed by surface and internal passivation layers. (f–l) Adapted with permission [4]. Copyright 2019, Springer Nature.

Related conversion‐anode studies on CuO and Fe_3_O_4_ likewise reported cycle‐ and voltage‐dependent interphase evolution, reinforcing that passivation in conversion electrodes is progressive rather than static [42, 43, 134]. Dachraoui et al. [133] directly visualized by *operando* liquid‐cell scanning transmission electron microscopy (STEM) that the interphase evolves from nanoparticle nucleation to island‐like growth and then to a thicker mosaic layer over the first several cycles (Figure 5a–e), rather than forming as a one‐step static film. These data matter here because they show that interphase growth in a conversion‐related interface is a cycling‐dependent process from the outset. Consistently, early‐cycle decomposition currents/peaks and high‐precision coulometry indicate that parasitic consumption can remain detectable well beyond the initial cycle even when capacity appears stable [114, 133, 135]. Under continued cycling, this dynamic interphase no longer acts only as a surface film; in conversion electrodes, it becomes coupled to the accumulation of internal passivation products, progressively shifting the dominant degradation mode from repeated surface reduction to a combined surface/internal transport barrier.

#### 5.1.2 Interphase Thickening and Impedance Rise as a Self‐Amplifying Process

SEI growth can become self‐reinforcing: once dynamic interphase evolution becomes coupled to the accumulation of both surface and internal passivation products in conversion electrodes, increasing interfacial/transport resistance raises polarization. It can prolong time spent at highly reducing potentials or under higher overpotential, which in turn promotes further electrolyte reduction and additional interphase thickening [4, 39, 136]. *Operando* diagnostics, such as stack‐pressure/volume tracking, have shown that SEI‐related irreversible expansion can proceed well beyond the first cycle and correlate with rising resistance [132]. A complementary magnetite study that combined XAS and XPS likewise showed that interphase formation and electrochemical reversibility are closely coupled, underscoring that passivation growth and reversibility loss should be interpreted together rather than inferred only from galvanostatic capacity fading [94].

Li et al. [4] further showed that the charged‐state microstructure of Fe_3_O_4_ after 100 cycles contains larger Fe‐containing bright regions and larger Li_2_O‐rich dark regions than after 3 cycles (Figure 5f–i), indicating accumulation of both conversion products and passivation‐rich domains. Their schematic interpretation in Figure 5j–l converts this microscopy evidence into a transport argument: as the surface layer and the internal Li_2_O‐rich passivation layer grow together, the effective diffusion length increases, and the access of both Li^+^ and electrons to active domains becomes progressively restricted. Accordingly, stabilization claims should be evaluated based on time‐resolved CE and round‐trip energy efficiency [126, 127], together with matched‐SOC resistance or impedance evaluation [25].

#### 5.1.3 Dissolution, Migration, and Redeposition Drive Cross‐Talk and Interphase Degradation

Cathode‐to‐anode cross‐talk should be treated as the transport of cathode‐derived degradation products to the negative electrode, where they can modify SEI chemistry, catalyze additional electrolyte reduction, and accelerate resistance growth [137, 138, 139, 140, 141, 142]. Classic Mn‐dissolution studies showed severe SEI degradation after Mn deposition on carbonaceous anodes [138, 139], while later mechanistic work linked this process to electrolyte/salt chemistry and LiPF_6_‐derived acidic species such as HF [140, 141, 142]. Accordingly, cross‐talk claims should be supported by evidence of dissolved‐metal deposition and linked to CE‐ and SOC‐matched impedance trends under matched conditions [138, 139].

#### 5.1.4 Protocol‐ and Condition‐dependence: Electrolyte Availability, Wetting, Formation Trajectory, and Electrode‐Level Nonuniformity

Interphase evolution is strongly conditioned by cell design and protocol variables, including E/C and the wetting/soaking state [76], as well as formation sequences (currents, holds, and cutoffs) that set the initial SEI chemistry/morphology and influence the subsequent resistance‐growth trajectory [37, 109]. In thick/porous electrodes, incomplete wetting and nonuniform ionic access can localize reaction and accelerate continuous decomposition; thus, comparisons should be made at matched areal loading/thickness/density with explicitly defined wetting and equilibration criteria prior to formation [17].

### 5.2 Mitigation Strategies

#### 5.2.1 Electrolyte Engineering to Suppress Continuous Electrolyte Reduction and Stabilize the SEI

Electrolyte engineering (salt/solvent/additive selection) aims to promote rapid passivation that suppresses continued electrolyte reduction while maintaining Li^+^ transport through the interphase [39, 136]. For an additive‐focused interpretation, FEC remains a reference additive for discussion of interphase formation because its behavior is not merely empirical. Shkrob et al. [44] showed that FEC undergoes a reductive decomposition pathway that differs from that of conventional carbonates, whereas *operando* studies on Fe_3_O_4_ showed that FEC‐based electrolytes form a more stable and compact interphase than EC‐based electrolytes [42]. For conversion anodes with surface renewal, additive effects should be evaluated using sustained CE and SOC‐matched resistance/impedance trends [126], rather than relying solely on first‐cycle SEI composition. When transition metal (TM) dissolution/cross‐talk is relevant, electrolyte strategies should also mitigate salt‐derived acidic species (e.g., HF from LiPF_6_ hydrolysis) [141] because they accelerate cathode corrosion and promote redeposition‐driven SEI degradation on the anode [138, 139]. Since LiPF_6_ hydrolysis and subsequent acid chemistry depend strongly on trace moisture and thermal history, TM‐dissolution/cross‐talk comparisons should report moisture control and thermal history explicitly [141, 143].

#### 5.2.2 Artificial Interphase and Surface Coatings for Interfacial Stabilization under Repeated Surface Renewal

Unlike Section 4.2.3, which focuses on first‐cycle lithium loss, the emphasis here is on long‐term interphase durability under repeated surface renewal. Artificial SEI layers and surface coatings can mitigate continuous decomposition by suppressing electron transfer to the electrolyte while maintaining Li^+^ transport [121, 124]. For conversion‐type anodes, coatings must tolerate large strain/volume changes and remain conformal; otherwise, interfacial delamination/cracking can reopen fresh surfaces and sustain continuous parasitic reactions [75]. Claims for interfacial stabilization in working conversion electrodes should therefore be supported by suppressed CE drift and slowed SOC‐matched resistance growth, together with cycling‐resolved surface‐sensitive evidence acquired at defined cycle number and SOC, rather than by single‐point post‐mortem chemistry alone [41, 42, 43]. More broadly, separator/interphase‐stabilization studies in conventional LIBs also reinforce the importance of maintaining structural and interfacial stability under practical conditions [144].

#### 5.2.3 Electrode Architecture and Mechanical Mitigation to Limit Crack‐Driven Surface Exposure and Current Localization

Particle and electrode architectures mitigate interphase instability indirectly by reducing crack‐driven fresh‐surface generation and suppressing reaction localization, rather than by directly altering SEI chemistry [75]. Representative examples include Mn_3_O_4_‐C composite microspheres with open three‐dimensional channels [145], CoS_2_ confined within an N, S‐co‐doped carbon nanotube hollow architecture [146], hollow Co_3_O_4_ particles [61], and Co2P quantum‐dot/carbon hybrids [50], all of which reduce repeated fresh‐surface generation by combining free volume with continuous conductive confinement. Their success should therefore be judged by slower CE drift and resistance growth, not merely by improved capacity retention.

#### 5.2.4 Mitigating Dissolution‐Mediated Cathode‐to‐Anode Cross‐Talk Using Electrolyte, Interfacial Barriers, and Separators

Cathode‐to‐anode cross‐talk driven by dissolved species can be mitigated by (i) suppressing dissolved‐species generation (e.g., reducing acid‐driven cathode corrosion), (ii) blocking migration with functional separators/interlayers, or (iii) limiting redeposition/reactivity at the anode surface. Additives that scavenge HF/acidic species or stabilize fluorine‐rich interphases can suppress TM dissolution and reduce redeposition‐driven SEI degradation [147, 148], while separator/interlayer concepts that impede TM‐ion transport can directly reduce deposition on the negative electrode [149]. Any cross‐talk mitigation claim should be supported by quantitative dissolved‐metal (and deposition) evidence and linked to improved CE and stabilized SOC‐matched impedance trajectories under matched conditions [138, 139].

### 5.3 Integrated Interpretation and Validation Checklist

#### 5.3.1 Integrated Interpretation

Collectively, interphase instability in conversion anodes should be treated as a transition from continued surface reduction to coupled surface/internal transport blockage, rather than as simple thickening of a static SEI. Repeated surface renewal sustains electrolyte reduction, later‐cycle passivation layers elongate the effective transport path, and cathode‐derived degradation products can further perturb the same anode interphase [137, 138, 139, 150]. Stabilization claims should therefore be anchored to time‐resolved CE, round‐trip energy efficiency, and SOC‐matched resistance trajectories rather than to capacity retention or endpoint surface spectra [25, 151].

Case 1. When capacity retention appears stable, but CE drifts or resistance rises.

Interpret the result as continued SEI growth and contact degradation rather than stable passivation, and support this interpretation with SOC‐matched resistance trends rather than a single late‐cycle spectrum [25, 132].

Case 2. When CE remains low or drifts despite modest early polarization.

Treat sustained parasitic reduction and incomplete passivation as dominant when CE remains low or drifts; nominal capacity stability can coexist with ongoing inventory and energy loss [37, 39, 109].

Case 3. When dissolution/cross‐talk is invoked as a driver of degradation.

Interpret the system as one in which cathode‐derived metal dissolution, transport through the electrolyte, redeposition on the anode, and additional electrolyte reduction occur together. Dissolved‐metal and deposition evidence should therefore be evaluated together with CE and SOC‐matched impedance trends [138, 139, 149].

#### 5.3.2 Validation Checklist

1. Judge stabilization from time‐resolved CE, round‐trip energy efficiency, and SOC‐matched resistance trends, not capacity alone: Report CE (or cumulative efficiency) together with round‐trip energy efficiency and impedance measured at matched SOC under a consistent protocol, because stable capacity can mask continuous lithium and electrolyte consumption [25, 151]. In conversion anodes, this is particularly important because the interphase can evolve progressively during cycling [42, 43]. Avoid inferring stabilization from endpoint electrochemical impedance spectroscopy (EIS) alone, because growth rates and resistance partitioning can be missed [132].
2. Distinguish interphase growth from internal passivation and contact‐driven resistance: When CE drift, impedance rise, or polarization growth is attributed to interphase instability, the interpretation should distinguish surface SEI thickening from internal passivation products, fracture‐driven fresh‐surface exposure, and loss of electronic contact [4, 39, 75]. This distinction is necessary because conversion electrodes can accumulate both surface and internal passivation layers during cycling, while chemo‐mechanical damage and carbon–binder‐domain degradation can produce similar resistance‐growth signatures [75, 92]. Therefore, interphase‐related claims should be supported by combining surface‐sensitive measurements with morphology‐ or contact‐sensitive evidence and SOC‐matched impedance tracking [25, 41, 94].
3. When interphase stabilization is claimed, include time‐resolved interphase evidence from working electrodes: Prefer *operando*, in situ, or cycle‐resolved surface‐sensitive tracking in working electrodes over single‐point post‐mortem spectra. When operando evidence is not available, post‐mortem samples should be harvested at defined cycle numbers and SOCs and interpreted together with CE and SOC‐matched resistance trends, because conversion‐anode interphases can evolve progressively with cycle number, SOC, and electrolyte chemistry rather than remaining static after formation [42, 43, 94].
4. When cathode‐to‐anode cross‐talk is invoked, quantify dissolution and redeposition: Measure dissolved metals and deposition evidence and connect them to improved CE and resistance trends; otherwise, cross‐talk remains an inference [138, 139]. Separator/interlayer claims should be evaluated as transport control of dissolved species, not morphology claims alone [149].

## 6 Large Volume Change, Particle Fracture, and Contact Loss

Chemo‐mechanical degradation is pervasive in conversion‐type anodes (metal oxides, sulfides, selenides, and phosphides) because conversion–reconversion repeatedly forms and decomposes metal–LiX nanocomposites (X = O, S, Se, and P) and reconstructs internal interfaces, generating large conversion strain and a time‐evolving electronic percolation network, often governed by the CBD [75]. These processes promote particle fracture/pulverization and progressive loss of electrical contact, which typically manifest as increased polarization/overpotential, broader voltage‐profile hysteresis, and spatially nonuniform utilization. Fracture‐driven fresh‐surface exposure and repeated SEI rupture/reformation can further sustain interphase growth and continuous electrolyte decomposition, accelerating impedance rise even when early capacity retention appears acceptable [39, 75].

Under practical areal loading and calendered density, particle‐scale damage couples strongly to electrode‐scale transport limitations and nonuniform electrolyte infiltration/wetting, so stress, reaction, and degradation localize spatially rather than remaining uniform [17]. Accordingly, mechanistic attribution should treat fracture/contact evolution and electrode heterogeneity as a coupled electro‐chemo‐mechanical problem rather than an active‐material‐only limitation [27]. Recent electro‐chemo‐mechanical reviews further argue that stress evolution, fracture, and transport heterogeneity should be treated as a single coupled problem across both particle and electrode scales, rather than as separable failure modes or as a purely active‐material issue [22, 23].

### 6.1 Origins and Representative Hypotheses

#### 6.1.1 Conversion‐Induced Strain and Stress–Electrochemistry Coupling

Conversion reactions generate multiple phases and internal interfaces with distinct molar volumes and mechanical properties, leading to stress accumulation as reaction fronts propagate and nanocomposite morphologies reorganize across cycles. Recent coupled electro‐chemo‐mechanical analyses suggest that stress can alter local chemical potentials, thereby modifying reaction and transport rates [155, 156]. In conversion systems, direct evidence further shows that controlling reaction‐front geometry and boundary conditions can suppress fracture and change electrochemical response, indicating that measured rate capability and cycling stability can be mechanically conditioned [91].

#### 6.1.2 Particle Fracture, Pulverization, and Fresh‐Surface Exposure

**FIGURE 6:**
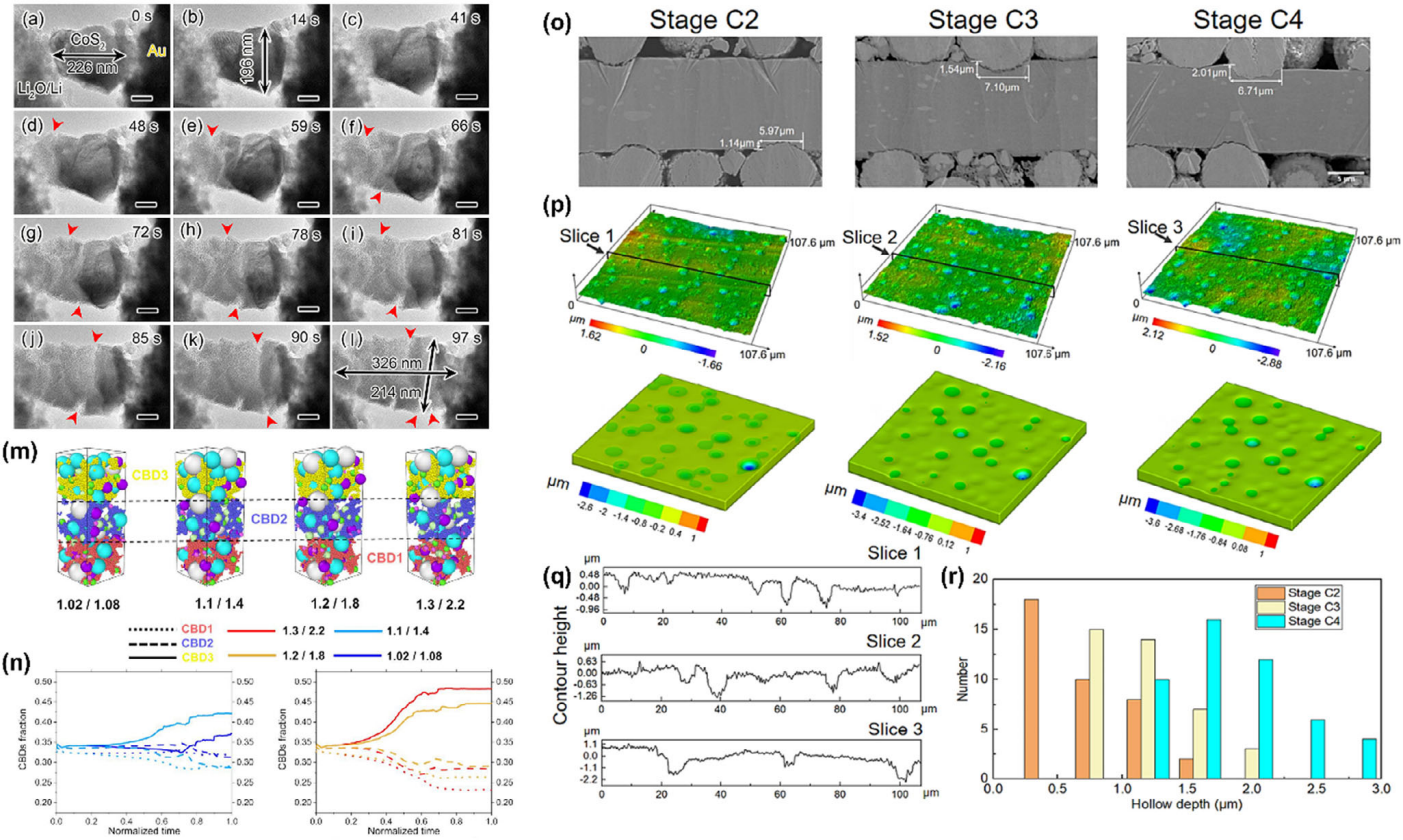
Chemo‐mechanical degradation across particle, composite‐network, and current‐collector length scales. (a–l) In situ TEM sequence of CoS_2_ lithiation showing side‐to‐side conversion, anisotropic expansion, crack initiation, and fracture development. Reproduced with permission [152]. Copyright 2014, American Chemical Society. (m,n) Three‐dimensional drying‐model prediction of carbon‐binder‐domain migration, showing how drying history redistributes CBD‐rich regions (CBD1/2/3) and creates heterogeneous conductive backbones before cycling begins. Adapted with permission [153]. Copyright 2021, Elsevier. (o) Cross‐sectional scanning electron microscopy (SEM) images of the particle–current collector interface at progressively stronger calendering stages (C2–C4), showing increasing particle intrusion and deformation of the current collector. (p) Reconstructed surface‐topography maps of the current collector after calendering, extracted from representative slices, showing the spatial distribution of surface depressions generated by particle indentation. (q) Contour‐height profiles along representative slices of the current collector surface, quantifying the magnitude and spatial variation of the depressions formed after calendering. (r) Hollow‐depth distribution of the current collector surface at different calendering stages, showing the progressive increase in both the number and depth of particle‐imprinted surface defects. (o–r) Adapted with permission [154]. Copyright 2023, Elsevier.

Su et al. [152] directly visualized by in situ TEM that CoS_2_ lithiation proceeds with anisotropic expansion and crack/fracture development during the reaction itself (Figure 6a–l). These images are important because they show that a fracture can form during a single conversion event, not only after long‐term cycling accumulation. Comparable in situ or *operando* studies on ZnO, RuO_2_, MoS_2_, and FeS_2_ likewise reported severe morphological reorganization during lithiation, indicating that fracture is a general consequence of conversion strain rather than a CoS_2_‐specific anomaly [91, 157, 158, 159, 160].

#### 6.1.3 Contact Loss and Electronic‐Percolation Breakdown in Composite Electrodes

In practical composite anodes, a major penalty arises from the degradation of the electronically percolating CBD and from the evolution of particle–CBD and particle–current collector contacts [67, 75, 161]. Three‐dimensional characterization, CBD‐network reviews, and chemo‐mechanical models show that CBD morphology/connectivity governs effective transport, local utilization, and stress transmission [68, 92, 162]. Drying and calendering can therefore pre‐imprint heterogeneity before cycling begins. Lombardo et al. [153] showed by three‐dimensional drying simulations that carbon–binder migration redistributes CBD‐rich regions before electrochemical testing (Figure 6m,n), while heterogeneous modeling and nanoscale imaging studies quantified local active‐material–CBD detachment, impedance rise, and particle‐size‐/rate‐dependent binder failure [163, 164, 165]. In this context, Figure 6m,n is not merely a processing detail; it shows that the conductive backbone can already be spatially heterogeneous before electrochemical degradation begins. Repeated volume change then amplifies this tendency toward electronic isolation and local overpotential growth [67, 68, 75].

#### 6.1.4 Electrode‐Level Heterogeneity Under Practical Loading: Densification, Wetting Limitation, and Interfacial Damage

Electrode densification from calendering and cycling reduces porosity and increases tortuosity, strengthening through‐thickness ionic‐transport gradients and increasing sensitivity to electrolyte availability [166]. Because conversion anodes already undergo evolving porosity and internal restructuring, insufficient wetting/infiltration can lock in depth‐wise utilization gradients and concentrate stress and reaction in a subset of the electrode [17, 27]. In anode manufacturing and translation, calendering has been shown to materially affect transport‐limited response and fast‐charge behavior in practical anode composites, reinforcing that density optimization is inseparable from accessibility control [166, 167]. Song et al. [154] combined cross‐sectional scanning electron microscopy (SEM), surface‐topography analysis, contour‐height profiles, and hollow‐depth statistics across progressively stronger calendering stages and showed that particle intrusion progressively deepens surface depressions and damages the current collector (Figure 6o–r). This is the current‐collector‐scale part of the same problem: the interface can inherit processing‐induced defects that later bias current constriction, local stress concentration, and mechanically amplified degradation. Microstructure‐resolved analyses further suggest that, in dense composite electrodes, transport‐driven reaction localization can create local stress concentration and current constriction, accelerating fracture and contact loss beyond what would be expected from single‐particle kinetics alone [168, 169].

### 6.2 Mitigation Strategies

#### 6.2.1 Particle and Interfacial Designs to Accommodate Conversion Strain

Particle and interfacial mitigation work by redistributing stress, providing free volume for breathing, or altering reaction‐front geometry to suppress stress concentration during conversion [75]. Representative examples include Fe_3_O_4_/Cu nano‐architectures [54], Fe_3_O_4_@Fe_3_C–C yolk–shell particles [53], multilayer CuO@NiO hollow spheres [60], carbon‐coated yolk‐shell V_2_O_3_ microspheres [59], hollow Co_3_O_4_ [61], Co_2_P quantum‐dot/carbon hybrids [50], FeS_2_ front‐shape engineering [91], and one‐dimensional chalcogenides such as V_2_Se_9_ [170]. As noted in Table 1, these particle‐ and interface‐engineering examples are used here to illustrate how free volume, confinement, or controlled reaction‐front geometry can mitigate conversion strain, rather than as universal proof of practical electrode durability. Their translation should be judged under matched loading/thickness/density, electrolyte availability, wetting/equilibration, and full‐cell or lean‐electrolyte conditions.

Particle‐size distribution should be optimized with the same trade‐off in mind. Large particles or broad distributions containing a large‐particle tail can develop stronger Li^+^ concentration gradients, nonuniform conversion fronts, and stress localization, which promote fracture, pulverization, and contact loss [75, 91]. However, simply minimizing particle size is not necessarily favorable, because excessive nanosizing increases specific surface area, electrolyte demand, first‐cycle lithium consumption, and continuous interphase growth [37, 39]. A narrow particle‐size distribution can be useful for isolating intrinsic particle‐size effects and reducing stress heterogeneity, whereas a controlled bimodal or moderately broad distribution may improve packing density and electrode processability if wetting and transport remain uniform. Therefore, particle‐size‐distribution optimization should report not only average size but also D10/D50/D90, specific surface area, tap density or electrode density, and relevant morphology or porosity descriptors. These descriptors should be interpreted together with CE, SOC‐matched resistance, and post‐cycling morphology/contact evidence, because apparent size benefits can otherwise reflect changes in surface area, packing, wetting, or conductive‐network integrity rather than intrinsic fracture resistance [164, 165, 171, 172].

Because particle downsizing, hollow structures, and high‐surface‐area frameworks can increase reactive surface area and electrolyte‐accessible interfaces, a convincing mitigation claim should demonstrate not only improved mechanical accommodation, but also stable CE and suppressed SOC‐matched impedance or resistance growth [37, 39].

#### 6.2.2 Composite Engineering to Preserve the Carbon–Binder Conductive Backbone

Composite engineering aims to maintain a stable electronic percolation network under repeated volume changes by optimizing binder chemistry and adhesion, conductive‐additive networks (e.g., carbon black/carbon nanotube/graphene hybrids), and CBD integrity to limit percolation loss and local current constriction [173]. Recent progress in polymeric binder chemistry reinforces this view by showing that binders can be designed as multifunctional components that control adhesion/cohesion, dispersion stability, mechanical resilience, electrochemical stability, electrolyte compatibility, and ionic/electronic transport [174]. Binder‐focused strategies should therefore be discussed by explicitly linking binder/CBD choices to resistance contributions and mechanical integrity, not only to capacity retention [67, 68]. To make this selection more explicit, Table 2 summarizes how conductive agents and binders can be chosen according to the dominant conversion‐anode penalty being targeted. The table builds on the carbon–binder‐domain, contact‐loss, conductive‐network, and polymeric‐binder design discussions in this review [67, 68, 92, 93, 153, 164, 165, 173, 174, 175, 176], and is intended as a practical selection guide rather than an exhaustive ranking of binder or conductive‐agent chemistries. When improved contact retention is claimed, at least one connectivity‐sensitive descriptor, such as 3D CBD morphology/connectivity or another quantitatively defined indicator, should be provided and related to polarization or impedance evolution [92, 93]. This requirement is important because carbon‐binder detachment depends on adhesion, particle size, viscoelasticity, and cycling rate [164, 165], and because processing can imprint CBD heterogeneity before cycling begins. As illustrated by Figure 6m,n, drying‐induced binder migration and calendering history can set the initial CBD distribution and thereby influence where contact loss initiates and how rapidly it propagates [153, 175, 176].

**TABLE 2:** Practical selection guide for conductive agents and binders in conversion‐type anodes.

Material/strategy	When it is useful	Main limitation or trade‐off	Recommended diagnostic check
Carbon black/particulate conductive additives	Provides local electronic contact and is useful when particles are small and electrode loading is modest.	Point‐contact networks can be disrupted by conversion strain, particle displacement, or binder migration.	Track SOC‐matched resistance and, where possible, CBD/contact morphology after cycling.
CNT or carbon‐fiber networks	Useful for large‐volume‐change materials and thicker electrodes because long‐range percolation can be maintained under strain.	Poor dispersion or excessive network stiffness can create heterogeneity, viscosity issues, or nonuniform porosity.	Report dispersion quality, electrode density/porosity, resistance evolution, and utilization uniformity.
Graphene/2D carbon frameworks	Can improve interfacial contact, confine particles, and provide flexible conductive pathways.	Restacking, increased surface area, and higher electrolyte demand can aggravate SEI growth and lithium consumption.	Pair capacity/rate gains with ICE, early CE, E/C ratio, and interphase or resistance tracking.
Hybrid conductive networks	Combining carbon black with CNTs, graphene, or porous carbon can balance local contact and long‐range electronic pathways.	Additional conductive phases can change porosity, tortuosity, slurry rheology, and wetting behavior.	Report formulation, loading/density, transport descriptors, and SOC‐matched resistance.
High‐adhesion or elastic binders	Useful when fracture, pulverization, or particle/CBD detachment dominates. Examples include carboxymethyl cellulose/styrene‐butadiene rubber‐, poly(acrylic acid)‐, alginate‐, polyimide‐, or self‐healing‐type binders depending on chemistry.	Excessive stiffness or swelling can induce stress concentration, delamination, or pore blockage.	Relate adhesion/cohesion or mechanical descriptors to morphology, CE, and resistance evolution.
Ion‐conductive or electrolyte‐compatible binders	Useful in thick or dense electrodes where ionic access and wetting are limiting.	High electrolyte uptake can improve wetting but may also cause binder swelling and conductive‐network disruption.	Report electrolyte uptake/swelling, wetting/equilibration protocol, E/C ratio, and rate behavior at matched saturation.
Conductive polymer binders	Can reduce reliance on separate conductive additives and help stabilize electronic pathways.	Electrochemical stability, mechanical robustness, and doping‐state stability must be verified under anode potentials.	Verify conductivity retention, electrochemical stability window, SOC‐matched impedance, and cycling morphology.

#### 6.2.3 Electrode Architecture, Drying, and Calendering Control for Uniform Utilization

Unlike Section 7.2, which focuses on thick‐electrode transport penalties themselves, the emphasis here is on chemo‐mechanically coupled localization: architecture, drying history, and calendering matter because they determine where fracture, contact loss, and fresh‐surface generation initiate at practical densities. Electrode architecture mitigates localization by distributing ionic/electronic pathways more uniformly across the thickness and by ensuring adequate wetting/infiltration at practical densities [27, 177]. Because drying and calendering can strongly shape electrode microstructure, mitigation should be implemented together with manufacturing controls that suppress carbon/binder segregation during drying and manage pore/connectivity evolution during calendering [153, 166, 175]. Any architecture, drying, or calendering claim should explicitly define electrode density/porosity and wetting/infiltration criteria, since accessibility‐limited regions can masquerade as mechanical failure or intrinsic active‐material instability.

#### 6.2.4 Current‐Collector Interface Management

Song et al. [154] showed that managing the particle–current collector interface, especially by limiting excessive particle intrusion during calendering and preserving collector‐surface integrity, can mitigate electronic constriction and delay polarization growth. Because this interface damage is introduced during manufacturing and can later be amplified during cycling, interfacial strategies should be evaluated using resistance/polarization trajectories under matched electrode density and wetting/equilibration conditions. The damage patterns in Figure 6o–r emphasize that this interface is not a passive geometric boundary: particle intrusion during calendering can predefine sites of electrical constriction and mechanical weakness, so current‐collector strategies should be evaluated as a chemo‐mechanical mitigation strategy rather than as a secondary manufacturing detail.

### 6.3 Integrated Interpretation and Validation Checklist

#### 6.3.1 Integrated Interpretation

Overall, chemo‐mechanical degradation in conversion anodes is best treated as a multiscale feedback loop linking particle fracture, conductive network degradation, current‐collector interface damage, polarization growth, and interphase evolution [39, 75, 178]. In other words, the electrochemical penalty usually attributed to mechanical degradation is rarely a particle‐only phenomenon. Mechanistic attribution, therefore, requires separating active‐material fracture from conductive network degradation and processing‐imprinted heterogeneity using SOC‐matched resistance tracking and connectivity‐sensitive evidence [25, 92]. A similar diagnostic distinction has recently been emphasized for silicon‐based alloying anodes, where degradation pathways are separated into active‐material degradation, electrode integrity loss, SEI instability, lithium trapping, and crosstalk [179]. Although silicon and conversion‐type anodes store lithium through different reaction mechanisms, this framework provides a useful diagnostic parallel because both systems couple large volume change, interphase renewal, contact loss, and electrode‐network degradation.

Case 1. When capacity fades slowly, but polarization grows steadily.

Interpret the result as progressive contact loss and resistance redistribution—such as CBD degradation or current constriction—rather than as worsening of the intrinsic reaction barrier. Validate this interpretation by correlating SOC‐matched resistance growth with a connectivity‐sensitive indicator [92, 93].

Case 2. When fracture indicators increase, and CE worsens.

Treat crack‐driven fresh‐surface exposure as coupled to sustained electrolyte reduction and interphase thickening; mechanical mitigation alone is insufficient unless it also suppresses leakage and resistance growth [39, 91]. The key validation is whether CE and SOC‐matched resistance stabilize together.

Case 3. When thick‐electrode performance collapses after densification.

Interpret the outcome as coupled densification/tortuosity increase and wetting limitation that induces through‐thickness reaction nonuniformity and mechanically accelerated damage in the most resistive zones [166, 167]. Control for infiltration/equilibration history to avoid accessibility limitations being misread as intrinsic mechanical instability [17].

#### 6.3.2 Validation Checklist

1. Separate particle fracture from conductive network degradation before assigning the dominant failure mode: Report drying and calendepoly(acrylic acid)ring conditions and any observed interfacial damage at the collector, because processing‐imprinted heterogeneity can dominate long‐term polarization growth [153, 180]. This is crucial to prevent claims of intrinsic active‐material durability when the observed behavior is actually dominated by conductive network degradation or microstructural heterogeneity.
2. Provide direct evidence for fracture/contact evolution when invoked: If fracture, pulverization, or contact loss is central to the mechanism, include direct microstructural evidence rather than relying on electrochemistry alone [22, 23]. When particle‐size or particle‐size‐distribution effects are used to explain improved mechanical durability, report distribution descriptors such as D10/D50/D90, specific surface area, and electrode or tap density, and connect them to post‐cycling morphology/contact evidence and SOC‐matched resistance evolution. Figure 6a–l illustrates the expected evidentiary standard.
3. Include a connectivity‐sensitive descriptor for CBD/percolation claims: When contact preservation is claimed, include a connectivity‐sensitive descriptor and relate it to resistance partitioning [92, 93]. Figure 6m,n illustrate why qualitative statements about dispersion or binding are insufficient.
4. Track polarization together with SOC‐matched resistance growth: Pair rate and polarization trends with impedance measured at matched SOC to determine whether performance drift is driven by contact degradation or interphase‐related resistance growth [25]. Endpoint spectra alone can miss the growth trajectory and bias interpretation. Figure 6o–r shows that processing‐imprinted collector damage can already bias later polarization growth, which is why the processing state must remain explicit in any chemo‐mechanical interpretation.
5. When thick‐electrode translation is discussed, show where degradation localizes: Provide at least one utilization‐uniformity or reaction‐distribution indicator across thickness when localization is part of the narrative [27]. This matters because localization can amplify mechanical damage and interphase growth even when average capacity looks acceptable [17].

## 7 Kinetic Limitations and Thick‐Electrode Penalties

Electrode‐scale transport and accessibility penalties become increasingly dominant as areal loading and electrode density are raised toward practical targets for conversion‐type anodes. In porous‐electrode theory, electrolyte concentration polarization and ohmic drops scale with the transport length and effective tortuosity [38], so thickness‐dependent polarization can emerge even when the intrinsic interfacial kinetics remain unchanged. Recent architecture/design reviews likewise argue that thick‐electrode limitations should be treated jointly through architecture, fabrication route, and electrolyte amount and wetting constraints, rather than through thickness alone, because nominally similar dense electrodes can differ substantially in how they trade energy density against infiltration and transport [181]. This general picture is consistent with experimental thick‐electrode studies showing thickness‐driven polarization growth, underutilization of active material, and practical energy–power tradeoffs as loading increases [182, 183, 184].

Accordingly, the measured rate capability of thick conversion anodes should be interpreted as a coupled outcome of (i) ionic transport through the electrolyte‐filled pore network, (ii) electronic transport through heterogeneous solid‐phase percolation, often governed by the CBD, and (iii) time‐evolving interfacial/contact resistances that shift the resistance partition during cycling [97, 185]. In practice, formation‐imprinted interfacial resistance growth can further bias thick‐electrode rate trends [40].

Under electrolyte‐lean conditions, incomplete infiltration and slow imbibition can render the wetted state (degree of saturation) time dependent and spatially nonuniform; early rate data can therefore reflect differences in wetting and electrochemically accessible active material, rather than intrinsic kinetics alone [63, 186], consistent with reports of strong wetting–heterogeneity coupling in thick electrodes [17]. Recent manufacturing‐focused wetting studies further show that separator wetting, electrode imbibition, and full‐cell filling are distinct but coupled processes whose timescales depend on microstructure, interface pathways, and filling conditions rather than on porosity alone [187, 188, 189].

### 7.1 Origins and Representative Hypotheses

#### 7.1.1 Ionic Transport Limitation and through‐Thickness Reaction Localization in Thick Electrodes

**FIGURE 7:**
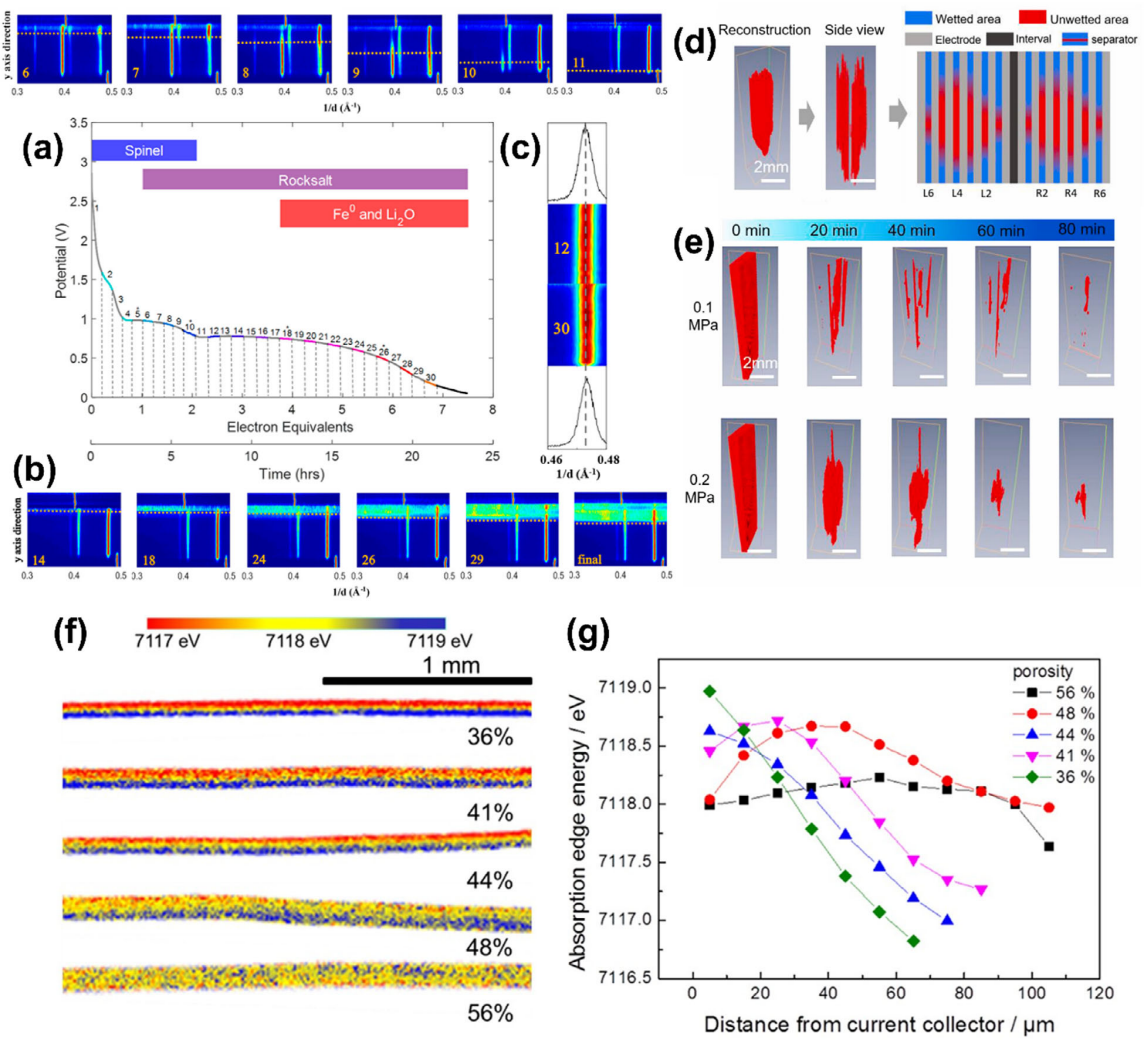
Thick‐electrode transport, wetting, and reaction‐distribution heterogeneity in conversion‐type electrodes. (a–c) *Operando* energy‐dispersive diffraction analysis of a magnetite electrode during lithiation, including representative through‐thickness diffraction maps at selected states, the electrochemical profile with the corresponding phase‐evolution guide (spinel → rocksalt → Fe^0^ + Li_2_O), and local diffraction‐signal evolution used to track reaction localization across electrode thickness. Reproduced with permission [26]. Copyright 2019, American Chemical Society. (d,e) In situ 4D visualization of electrolyte wetting heterogeneity, showing the spatial distribution of wetted and unwetted regions and their time‐dependent evolution under different applied pressures. Adapted with permission [32]. Copyright 2023, Elsevier. (f,g) Cross sectional Fe K‐edge absorption‐energy mapping and depth‐dependent edge‐energy profiles for composite electrodes with different porosities, showing that reaction distribution depends strongly on electrode microstructure and effective transport pathways. Reproduced with permission [31]. Copyright 2016, Springer Nature.

Tortuosity‐driven ionic transport limitation increases effective ionic resistance and steepens electrolyte concentration gradients as electrode thickness and areal capacity increase, leading to thickness‐dependent polarization even when intrinsic charge‐transfer kinetics are unchanged [66, 97]. *Operando* energy‐dispersive diffraction by Bruck et al. [26] directly showed that active‐phase evolution in a magnetite electrode becomes nonuniform across the thickness during lithiation (Figure 7a–c), demonstrating that reaction progress can become spatially heterogeneous even when the active chemistry is unchanged. Therefore, thick‐electrode polarization depends not only on how fast the average electrode responds, but also on where the reaction is concentrated through the electrode thickness. Because tortuosity governs effective transport in the pore network (e.g., *κ*_eff_ and *D*_eff_), it should be measured or estimated rather than assumed; impedance‐based methods and their validation against microstructure‐based approaches provide practical ways to report comparable transport descriptors across studies [29, 30, 190]. Microstructure‐resolved modeling and spatially resolved experiments further show that anisotropic pore networks, CBD topology, local tortuosity inhomogeneities, and charge‐distribution gradients can all bias thick‐electrode transport, so a single porosity value is insufficient for comparison [69, 92, 191]. Kehrwald et al. [192] and Liu et al. [193] reinforce this caution from a spatial perspective by showing local inhomogeneities in tortuosity and charge‐distribution gradients in working electrodes.

#### 7.1.2 Incomplete Wetting and Electrolyte Availability as Practical Accessibility Limiters

Incomplete wetting and limited electrolyte availability can dominate early utilization and the measured rate response in thick electrodes because the electrochemically active region expands only gradually and nonuniformly as infiltration proceeds [63, 74, 186]. Under limited electrolyte amount or incomplete wetting, ionic gradients can shift the dominant reaction region toward the separator side and suppress utilization in the electrode interior [17, 69]. Time‐ and spatially resolved wetting studies further show that electrolyte infiltration can remain incomplete and heterogeneous under practical filling or equilibration conditions (Figure 7d,e) [32]. Such wetting heterogeneity can change the electrochemically accessible volume and therefore distort early rate or utilization measurements.

Imaging and time‐resolved wetting studies also show that partial saturation can persist through thickness, while compression, pore‐size distribution, and temperature affect infiltration kinetics [28, 32, 194, 195]. Shodiev et al. [28] further showed that, even at fixed chemistry, different mesostructures can exhibit markedly different saturation‐versus‐time trajectories, indicating that electrolyte accessibility is architecture‐dependent and evolves over experimentally relevant timescales. Anode–separator interface studies likewise suggest that wetting can be governed more strongly by interface‐controlled pathways than by porosity alone [196]. Therefore, rate comparisons should distinguish faster wetting/saturation [28, 171, 172, 197] from improved steady‐state ionic transport at comparable saturation [29, 30, 97].

#### 7.1.3 Coupled Ionic–Electronic Resistance and Current‐Pathway Heterogeneity

When ionic gradients interact with heterogeneous electronic pathways, contact resistance, and interfacial resistances, reaction can localize and generate current constriction, increasing local overpotential and accelerating degradation where current density concentrates [185, 198]. Cross sectional reaction‐distribution mapping provides direct evidence for this effect. Orikasa et al. [31] showed that Fe K‐edge absorption‐energy maps and depth‐dependent edge‐energy profiles vary strongly with electrode porosity (Figure 7f,g), indicating that reaction distribution in composite electrodes is governed by effective ionic/electronic transport pathways rather than by the intrinsic active‐material reaction alone. Because electronic transport and current pathways can also be strongly influenced by CBD morphology/connectivity [67], kinetic‐improvement claims in thick electrodes should be checked by pairing rate data with matched‐SOC resistance tracking under a consistent protocol and at least one utilization‐ or reaction‐distribution indicator [25, 31].

### 7.2 Mitigation Strategies

#### 7.2.1 Electrode Architecture Design for Improved Ionic Transport in Thick Electrodes

Electrode‐architecture design in thick electrodes aims to reduce tortuosity‐limited ionic transport and concentration polarization by engineering pore connectivity, anisotropy, and graded structures [27, 199]. Bi‐tortuous designs, laser‐structured electrodes, and more recent bimodal microstructure engineering strategies all exemplify this approach [200, 201, 202]. Gradient active‐material distributions and tortuosity‐engineered architectures provide additional examples of electrode‐architecture control that shortens effective ion‐transport paths in thick electrodes [203, 204]. Their benefit should be demonstrated at the target thickness and density using a quantified transport descriptor rather than inferred solely from rate data [29]. Where electrolyte infiltration is part of the claimed benefit, infiltration acceleration should be distinguished explicitly from steady‐state transport improvement [28]. Related thick‐electrode studies further show that tortuosity gradients can localize lithiation through thickness [205].

#### 7.2.2 Electrode Densification with Preserved Ionic Accessibility and Wetting

Densification increases volumetric energy density but often increases tortuosity and slows infiltration, so mitigation focuses on preserving ionic connectivity while minimizing drying‐ and calendering‐induced heterogeneity [166, 206]. The density–accessibility tradeoff is not necessarily monotonic, since light calendering can initially improve wetting before stronger compaction slows it again [207]. Saturation‐versus‐time curves, neutron‐radiography filling studies, and calendering‐dependent fast‐charge analyses therefore all support the same conclusion: densification should be evaluated using infiltration‐sensitive descriptors under electrolyte‐lean conditions rather than porosity alone [28, 167, 188, 189].

#### 7.2.3 Mitigating Reaction Nonuniformity by Stabilizing Resistance Distribution and Current Pathways

Stabilizing the resistance distribution suppresses current constriction arising from coupled ionic gradients, electronic percolation heterogeneity, and contact/interfacial resistances [161, 185, 198]. Practical approaches include continuous conductive networks and controlled CBD morphology, which minimize contact resistance and maintain electronic continuity across the electrode thickness [67]. In thick electrodes, binder chemistry also contributes to current‐pathway stabilization because binder‐controlled dispersion, swelling, electrolyte compatibility, and conductive‐additive interaction can reshape both ionic access and electronic percolation [174]. Accordingly, the conductive‐agent and binder choices summarized in Table 2 should be interpreted not only as formulation variables, but also as practical tools for stabilizing current pathways and suppressing reaction nonuniformity in thick conversion electrodes.

As emphasized in impedance‐method papers and localization studies, attribution should rely on SOC‐matched resistance tracking with a transparent protocol and, where possible, spatial utilization diagnostics, because conductive network changes can mimic kinetic enhancement by redistributing current and accessed volume; when EIS is used, the interpretation should also state whether the fitted feature is assigned to bulk, interphase, charge‐transfer, or diffusion‐related resistance [25, 31, 208, 209].

#### 7.2.4 Evaluation and Processing under Electrolyte‐Lean Conditions with Explicit E/C and Wetting Control

Controlled electrolyte amount and wetting history are enabling conditions for thick‐electrode interpretation. Reporting electrolyte amount (or E/C) together with a defined wetting/equilibration protocol reduces misattribution of wetting‐controlled utilization as transport or kinetic gains [63, 74].

Because infiltration can remain incomplete on manufacturing‐relevant timescales and depends strongly on microstructure and compression state, wetting criteria (time, temperature, pressure/vacuum steps) should be specified and held constant across comparisons [186, 194]. Formation steps and cutoff logic should also be fully specified because formation imprints the early resistance trajectory and can shift the accessible utilization window in thick electrodes [38, 40].

### 7.3 Integrated Interpretation and Validation Checklist

#### 7.3.1 Integrated Interpretation

In thick conversion electrodes, the observed rate limitation is usually a coupled transport‐and‐accessibility problem rather than a single intrinsic charge‐transfer barrier [66, 196]. Reaction nonuniformity through the electrode thickness [26], incomplete electrolyte infiltration [28], and changes in resistance partition during cycling [25, 198] can all bias the measured rate response before any change in intrinsic active‐material kinetics is invoked. Changes in rate capability should therefore be interpreted in the context of the expected transport‐length dependence of polarization under matched areal loading, density, and electrolyte amount/wetting state [27, 64, 181]. High‐rate capacity measured in a short laboratory protocol should not be equated directly with practical power capability, because deliverable power is state dependent and must be interpreted under explicit SOC, temperature, aging, and voltage constraints [210].

Case 1. When thin‐electrode rate capability is strong, but thick‐electrode performance collapses.

Interpret this result as being dominated by transport gradients. Under load, thickness‐dependent concentration polarization suppresses utilization in the electrode interior and shifts the main reaction region toward the separator side [66]. Verify using thickness scaling and transport descriptors and confirm that the result is not simply a wetting‐induced bias [69].

Case 2. When early thick‐electrode performance is promising, but polarization drift accelerates with cycling.

Interpret this pattern as a shift in the resistance contribution of the electrode over time, driven by interfacial/contact growth and/or evolution of the conductive network [67]. Compare resistance at matched SOC and support claims with at least one conductive network‐ or utilization‐sensitive indicator [25].

Case 3. When outcomes depend strongly on electrolyte amount, wetting time, or formation details.

The observed rate response should be interpreted in the context of electrode accessibility and testing protocol, rather than being attributed solely to intrinsic material stability. In particular, differences in electrolyte wetting/active‐material accessibility and resistance evolution established during formation can dominate the measured behavior [63]. To avoid misattribution, the accessibility state should be defined explicitly, and cutoff criteria and formation procedures should be kept consistent across design comparisons [32, 38].

#### 7.3.2 Validation Checklist

1. Demonstrate thickness/loading scaling for thick‐electrode claims: Report performance across a thickness/density (or areal loading) series, because transport gradients and polarization scale strongly with geometry [66]. The shared reporting variables, including electrolyte amount, wetting/equilibration history, formation protocol, and matched‐SOC resistance comparison, are defined in Section 2; the penalty‐specific question here is whether the claimed improvement persists as transport length and areal loading increase.
2. Quantify transport descriptors instead of assuming Bruggeman‐like behavior: Report tortuosity and, where direct measurement is not possible, state the estimator used. Where possible, also report effective transport parameters using stated methods, because tortuosity is a first‐order control of concentration polarization in thick electrodes [29, 30]. This prevents interpreting thickness‐driven utilization loss as intrinsic kinetics. Figure 7a–c reinforces why quantified transport descriptors are needed: reaction nonuniformity can emerge through thickness even when the chemistry is unchanged.
3. Make the accessibility state explicit for electrolyte‐lean electrodes: Define the electrolyte amount and wetting/equilibration protocol, because incomplete infiltration can dominate the early rate response and strongly affect the fraction of the electrode that is effectively accessible to ionic transport [187, 188, 189, 196]. Figure 7d,e shows why this accessibility state must remain explicit.
4. Pair rate data with SOC‐matched resistance tracking: Use impedance measured at matched SOC to determine whether resistance partitioning stabilizes over cycling, since thick‐electrode rate limits often evolve rather than remain fixed [25].
5. Include at least one utilization‐uniformity indicator through thickness: Provide a reaction‐distribution or utilization‐uniformity indicator to demonstrate suppressed localization rather than a simple shift in the electrochemically active region [31, 69]. Figure 7f,g provides representative reaction‐distribution indicators for evaluating localization across electrode thickness.

## 8 Conclusion and Outlook

This review emphasizes that the performance of conversion‐type anodes is governed by the coupled evolution of reaction pathways, interfaces, and composite‐electrode transport, rather than by stoichiometric reversibility alone. Across the literature, five penalties recur and frequently co‐amplify one another: voltage hysteresis reduces energy efficiency; low ICE depletes cyclable lithium; dynamic and continuously growing interphases sustain parasitic consumption and resistance rise; chemo‐mechanical damage renews reactive surface area while disrupting electronic percolation; and thick‐electrode transport gradients promote reaction localization. A key implication is that many mitigation strategies inherently modify multiple variables at once, so apparent improvements in voltage profiles, capacity retention, or rate capability can arise from redistributed utilization or shifted accessibility rather than a true reduction of the targeted intrinsic barrier.

Progress, therefore, requires evaluation and reporting frameworks that make claims falsifiable under realistic conditions. Studies should explicitly define electrode design and processing state, including areal loading, thickness, density/porosity, electrolyte availability such as E/C, wetting/equilibration protocol, and the complete formation sequence. Mechanistic attribution should be anchored to time‐resolved CE and energy‐efficiency trends, resistance or impedance evaluated at matched SOC, and, when pathway changes are claimed, phase‐ or intermediate‐sensitive evidence together with at least one indicator of utilization uniformity across the electrode. These minimum diagnostics should help separate intrinsic conversion/reconversion pathway effects from electrode‐level accessibility, resistance, interphase, and formation‐history effects.

Looking forward, a major challenge is to diagnose coupled degradation in working thick conversion electrodes without reducing the problem to a single endpoint measurement. Future studies should combine operando or time‐resolved diagnostics with practical electrode formats to track reaction localization, interphase evolution, resistance growth, and chemo‐mechanical damage in the same cell context. Spatially resolved phase/reaction mapping, cycling‐resolved interphase analysis, pressure or volume‐change tracking, and SOC‐matched impedance under controlled electrolyte amount and wetting history would be particularly useful for determining whether a mitigation strategy changes the intrinsic reaction pathway or primarily redistributes utilization.

A second priority is to translate nanostructured and hollow conversion‐anode designs from proof‐of‐concept half cells to high‐loading, electrolyte‐lean, and full‐cell formats. Such studies should report loading, E/C ratio, wetting history, CE, resistance evolution, and utilization uniformity together, because the same high surface area and porosity that improve apparent rate capability can also increase electrolyte demand, first‐cycle lithium consumption, and continuous interphase growth. Finally, microstructure‐level optimization, including data‐driven or physics‐informed approaches, should treat particle‐size distribution, pore architecture, tortuosity, carbon–binder‐domain connectivity, calendering density, and formation protocol as coupled design variables rather than independent optimization targets.

## Conflicts of Interest

The authors declare no conflicts of interest.

## Data Availability

Data sharing not applicable to this article as no datasets were generated or analysed during the current study.
